# Unveiling the interaction profile of rosmarinic acid and its bioactive substructures with serum albumin

**DOI:** 10.1080/14756366.2020.1740923

**Published:** 2020-03-23

**Authors:** Christina Papaemmanouil, Maria V. Chatziathanasiadou, Christos Chatzigiannis, Eleni Chontzopoulou, Thomas Mavromoustakos, Simona Golic Grdadolnik, Andreas G. Tzakos

**Affiliations:** a Department of Chemistry, Section of Organic Chemistry and Biochemistry, University of Ioannina, Ioannina, Greece; b Department of Chemistry, National and Kapodistrian University of Athens, Athens, Greece; c Laboratory for Molecular Structural Dynamics, Theory Department, National Institute of Chemistry, Ljubljana, Slovenia

**Keywords:** Rosmarinic acid, caffeic acid, salvianic acid, serum albumin, STD-NMR

## Abstract

Rosmarinic acid, a phytochemical compound, bears diverse pharmaceutical profile. It is composed by two building blocks: caffeic acid and a salvianic acid unit. The interaction profile, responsible for the delivery of rosmarinic acid and its two substructure components by serum albumin remains unexplored. To unveil this, we established a novel low-cost and efficient method to produce salvianic acid from the parent compound. To probe the interaction profile of rosmarinic acid and its two substructure constituents with the different serum albumin binding sites we utilised fluorescence spectroscopy and competitive saturation transfer difference NMR experiments. These studies were complemented with transfer NOESY NMR experiments. The thermodynamics of the binding profile of rosmarinic acid and its substructures were addressed using isothermal titration calorimetry. *In silico* docking studies, driven by the experimental data, have been used to deliver further atomic details on the binding mode of rosmarinic acid and its structural components.

## 1. Introduction

Nature operates through recycling of basic units to build structural and functional complexity. For instance, simple aminoacid building blocks are merged via the ribosomal machinery to build advanced function. This assembling is also observed in the structures of plant secondary metabolites such as oleuropein and oleocanthal, both based on the structures of tyrosol and elenolic acid. Another example is rosmarinic acid that is composed of two building blocks, salvianic acid and caffeic acid. These examples illustrate the efficiency of nature to develop new chemical space through combining available building blocks. A question that exists is whether the isolated building blocks or the conjugated forms could deliver higher potency to their interaction potential with proteins. To get a first glimpse on this we focussed on serum albumin and explored whether the binding profile of rosmarinic acid can be deconvoluted to its isolated components or a more optimal profile could be achieved in this higher structural complexity.

Serum albumins are found in liver and plasma and their main role is to deliver through the circulatory system numerous drugs, hormones as well as metabolites in the body^1^. Bovine serum albumin (BSA), is a 583 amino acid protein that belongs to the large family of serum albumins^2^. Of great importance is that BSA, as well as all the other serum albumins, consists of a number of binding sites, each of which is suitable for interaction with a different group of ligands. The most known binding sites for drugs are Sudlow sites I and II. Sudlow site II is characterised by strong hydrophobic interactions with small molecular weight containing aromatic rings or negative charge ligands. Drugs like ibuprofen, diazepam, as well as the aminoacid l-tryptophan are well known site markers for this binding site^3^. Conversely, Sudlow site I is characterised by low selectivity, and thus, numerous ligands with a structural variety (aromatic rings, fatty acid moieties, etc.), can bind to this site with high efficiency. Known drugs as site markers for this binding site of BSA are warfarin, aspirin and sulfonamide^3^. Ligands of different molecular weights like fatty acids^4^^,^^5^, flavonoids, drugs^6^^,^^7^ or even cationic metals^8^, have the potential to interact selectively with BSA at specific binding sites. This potential of BSA has led to many biophysical studies to evaluate the interaction mode between BSA and a potent ligand. Competitive experiments with known site markers, like ibuprofen, warfarin or tryptophan can reveal the specificity of the binding site for each ligand. Due to the efficient structural similarity to human serum albumin (HSA), at a percentage of 75%, as well as, a high similarity to the ligand binding pockets of HSA, BSA is widely used as a template to evaluate the interaction profile to the different binding pockets of serum albumin^2^.

Rosmarinic acid is a polyphenolic ester of hydroxycinnamic acids family and can be found in abundance in the plant kingdom. Its main source is *Rosmarinus officinalis*, of Lamiaceae family, although, it can be found in great amounts in basil, mint, lemon balm and other sources^9^^,^^10^. Rosmarinic acid has been widely studied for its bioactive and pharmacological properties as antioxidant and anti-allergic agent, oxidation inhibitor of low density lipoprotein, murine cell proliferation inhibitor and cyclooxygenase inhibitor^11^. Its use as antioxidant agent in foods^12^ and as scent in cosmetics^13^ is also of great importance.

As an ester of caffeic acid and (R)-(+)3–(3,4-dihydroxyphenyl) lactic acid, rosmarinic acid consists of two structural moieties: caffeic acid and (R)-(+)3–(3,4-dihydroxyphenyl) lactic acid. (R)-(+)3–(3,4-dihydroxyphenyl) lactic acid, known as salvianic acid, a natural compound which is not yet well defined and characterised. Although, there are chemical methods for producing salvianic acid, their yield remains low^14–17^. The most common synthetic procedures for salvianic acid involve its precursor 3,4-dihydroxybenzaldehyde, and, after numerous reaction steps, the obtained yield is very low while is produced a large amount of efflux^15^^,^^18^^,^^19^. An alternative method is its isolation from *Salvia miltiorrhiza* where salvianic acid is the active phytochemical substance and its extraction results to low yields as well^20^^,^^21^. Finally, another way of producing salvianic acid is its hydrolysis from rosmarinic acid or other natural products. Both the chemical and enzymatical methods have their own limitations. The chemical hydrolysis suffers from low yields while the enzymatic hydrolysis may be more efficient, due to high regioselectivity of the enzyme, although, more expensive.

In the present work, in order to avoid all aforementioned drawbacks we have attempted several conditions to achieve the maximum recovery of salvianic acid with methanolysis of rosmarinic acid in mild conditions. With this method not only we minimised the production of side products but the unreacted material can be further used or be hydrolysed again offering a “green approach” to this method taking in consideration that there are no side products or waste.

**Scheme 1.**
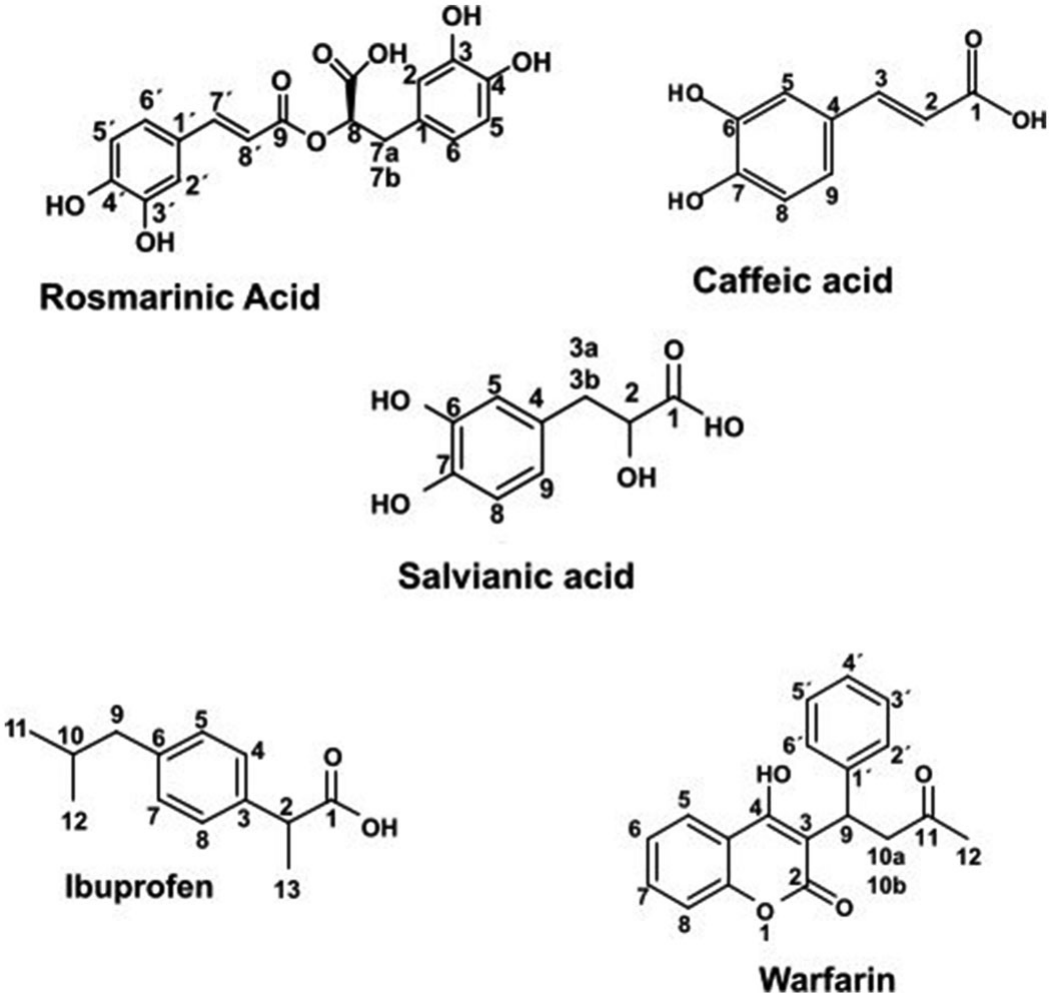
Structures of the studied compounds: rosmarinic acid, caffeic acid, salvianic acid, ibuprofen and warfarin.

In addition, in the present work, the interaction profile of BSA with rosmarinic acid and its substructure components have been revealed. Saturation transfer difference (STD) NMR has been used to unveil the binding profile of rosmarinic acid and its bioactive components with BSA. Furthermore, competitive STD NMR experiments have also been recorded with established site markers to specify the binding site of BSA to which each ligand interacts (Scheme 1).

Transferred-NOESY (tr-NOESY) experiments were recorded to define the conformation of the studied molecules in the BSA bound state. The binding affinities of rosmarinic acid and its two subscaffolds were estimated through isothermal titration calorimetry (ITC) while the derived thermodynamic parameters revealed the nature of the intermolecular forces involved in each interaction.

Molecular docking was applied as a complementary technique to provide a valuable insight on the BSA binding architecture.

## 2. Material and methods

### 2.1. Synthesis of salvianic acid

Salvianic acid was obtained using as starting material the natural product rosmarinic acid which was purchased from Sigma Aldrich. To achieve the hydrolysis of this molecule an optimised alteration of a very simple and efficient method of alkaline hydrolysis (methanolysis) was applied as described in our previous article^22^.

In particular, a methanolic solution of 1 M NaOH was added to a solution of the rosmarinic acid (102 mg, 0.28308 mmol) in CH_2_Cl_2_/MeOH (9:1, v/v, 13.4 mL). The mixture was stirred at 30 °C for 4 h and a large amount of olive-green precipitate was formed. The solvent was removed under vacuum, the residue was diluted with water and the aqueous solution was cooled and acidified with 1 N HCl until pH reached 3–4. The acidified aqueous phase was lyophilised and the saturated mixture was subjected to preparative HPLC chromatography to isolate the desired product (Supplementary Figure S1). A Jupiter 10 μm Proteo 90 A (250 × 21.2 mm) column was used while the mobile phase consisted of MeOH–H_2_O containing 0.1% TFA. A gradient elution (40–100% MeOH) was applied with 20 mL/min flow rate for 20 min and the detection was set to 280 nm. Salvianic acid was eluted at a retention time of 12.6 min, resulting to 12.4 mg of salvianic acid with a 22.1% yield.

^1^HNMR of salvianic acid (DMSO-*d*_6_, 500 MHz) (Supplementary Figure S2): δ(ppm) = 12.35 (s_wide_, 1H, 1-OH), 8.62 (s_wide_, 2H, 3′,4′-OH), 6.61 (d, J = 6.25 Hz, 1H, 5′-H), 6.58 (s, 1H, 2′-H), 6.44 (d, J = 8.28 Hz, 1H, 6′-H), 4.02 (m, 1H, 2-H), 2.76 (dd, J = 4.73 Hz, 5.01 Hz, 1H, 3-Ha), 2.59 (dd, J = 7.79 Hz, 8.07 Hz, 1H, 2-Hb).

^13^CNMR of Salvianic acid (DMSO-*d*_6_, 500 MHz) (Supplementary Figure S3): δ(ppm) = 116.9(C3´), 115.1(C4´), 116.5(C5´), 114.96(C2´), 119.88(C6´), 72.12(C2), 39.4(C3), 129.1(C1´), 143.5 (C1).

### 2.2. Fluorescence spectroscopic studies

The interaction between BSA and each of the three phenolic acids was examined through steady-state fluorescence spectroscopy studies being performed and analysed as described in previous works^23^^,^^24^. The fluorescence spectra were recorded on a Perkin Elmer LS-55 spectrofluorometer at room temperature using a 1.0 cm quartz cuvette. Gradual concentrations of rosmarinic, caffeic or salvianic acid (0–20 μΜ) were titrated into a BSA solution (2 μM). BSA was dissolved in PBS buffer (0.01 M, pH = 7.4) and the acids were dissolved in DMSO. The excitation wavelength for BSA was set to 285 nm and the maximum emission wavelength was occurred at approximately 350 nm. Both emission and excitation slits were 7 nm. The fluorescence data were, also, analysed by the Stern–Volmer equation:

$$F_{0}/F=1\;+K_{\text{SV}}[Q]=1+k_{q}\tau_{0}\left[Q\right],$$

where *F*_0_ and *F* are the maximum fluorescence intensities of the protein in the absence and presence of the ligand, respectively. The Stern–Volmer quenching constant for BSA is represented by *K*_SV_ and [*Q*] is the concentration of the ligand. *K*_q_ is the quenching rate constant for BSA and *τ*_0_, the fluorescence lifetime of BSA in the absence of a quencher was considered equal to 10^−8 ^s. The *F*_0_ and *F* values were corrected to eliminate the inner filter effect at the excitation (285 nm) and emission wavelengths (350 nm) caused by the low absorbance of the three phenolic acids at the specific wavelengths, using the equation^25^:

$$F_{\mathrm{cor}}=F_{\mathrm{obs}}{*\;10}^{-\left(\varepsilon_{\lambda \mathrm{exc}}+\varepsilon_{\lambda \mathrm{em}}\right)*l*L_{o}},$$

where $F_{\mathrm{obs}}$ is the maximum measured fluorescence of BSA and $F_{\mathrm{cor}}$ the corrected value of the maximum fluorescence of BSA; *ε_λ_*_exc_ or *ε_λ_*_em_ are the molar extinction factors of the ligand at the excitation and emission wavelengths of BSA; *L*_o_ is the total concentration of the bound and unbound ligand; and *l* is the path length in the measuring cell.

### 2.3. NMR experiments

#### 2.3.1. STD experiments

NMR samples for STD experiments were prepared in a 99.9% D_2_O buffer of 10 mM PBS, pH = 7.4. First, stock solutions of the ligands, rosmarinic acid, caffeic acid and salvianic acid were prepared in DMSO-*d*_6_. The concentration of the ligands in the NMR tube (600 μL) was 2 mM and the concentration of the protein (BSA) in the NMR tube was 50 μΜ, leading to a total ratio of the complex ligand–protein 40:1. Samples were subjected to STD experiments at 25 °C.

STD NMR experiments were recorded on Bruker AV 500 MHz spectrometer (Bruker Biospin, Rheinstetten, Germany) using the Topspin 2.1 suite. The spectral width of the spectra was 6009.615 Hz. Pulse sequences provided in Bruker libraries of pulse programmes were used. Relaxation delay was set to 1.5 s. Selective on-resonance irradiation frequency was set to 1.6 ppm with saturation time of 2 s. Selective saturation was achieved by a train of 50-ms Gauss-shaped pulses separated by a 2-ms delay. The duration of the presaturation of 2 s was adjusted using *n* = 16 cycles. Off-resonance irradiation frequency for the reference spectrum was applied at 20 ppm. Water suppression was achieved with excitation sculpting. Spectra were zero filled twice and the line broadening function of 3 Hz was applied. Then, STD experiments were performed, based on the basic method of STD NMR experiments^26^^,^^27^.

#### 2.3.2. STD ligand epitope mapping experiments

The STD experiments for the ligand epitope mapping were recorded on Agilent Technologies DD2 600 MHz Spectrometer (NMR Center, National Institute of Chemistry, Slovenia), using a cryoprobe, at 25 °C. The NMR samples were prepared in buffer 100% D_2_O, containing 10 mM Tris (98% *d*_11_). The pH was adjusted to 7.4, with the addition of DCl or NaOD. The reason we used Tris-*d*_11_ buffer for the STD NMR experiments, as well as the following tr-NOESY experiments, is that we are able to obtain a high quality NMR spectra with sufficient S/N ratio for quantitative analysis. Tris is also suitable for the water suppression experiments, as there are no signals of Tris-*d*_11_, near the water peak. However, all the NMR experiments which have been recorded by using phosphate buffer and Tris-*d*_11_, are the same^28^. First, one stock for each ligand (rosmarinic acid, caffeic acid, salvianic acid) and a stock of BSA solution were prepared in Tris-*d*_11_ 10 mM buffer. The samples were added in an NMR tube of total volume 600 μL. The concentration of each ligand was 2 mM, while the concentration of BSA was 20 μM, leading to a total ligand: protein ratio of 100:1.

The STD ligand epitope mapping experiments^26^ were acquired with 600 Hz spectral width, with 16,384 data points, 4096 scans and a relaxation delay of 2.5 s. The short protein saturation time of 0.22 s was used, selected according to the shortest ligand ^1^H longitudinal relaxation time (T_1_) determined in the presence of the protein to avoid the influence relaxation on STD amplification factors^26^. The ^1^H T_1_ values of ligands ranged from 0.44 s to 2 s. Selective saturation was achieved by a train of 50 ms long Gauss-shaped pulses separated by 1 ms delay. Water was suppressed via excitation sculpting. The on—resonance selective saturation of the protein was set to 0.81 ppm, more than 1000 Hz, away from the position of protons resonances of the ligands. Off-resonance irradiation was applied at 30 ppm. Subtraction of on- and off-resonance spectra was performed internally via phase cycling. The reference spectrum was recorded with the off-resonance irradiation at 30 ppm. Spectra were zero-filled twice and apodized by an exponential line broadening function of 3 Hz.

The STD amplification factor (STD_AMP_) was calculated by multiplying the ratio of STD signal intensity (I_STD_) and the intensity of the corresponding signal in the reference spectrum (I_0_) with the molar ratio of ligand in excess relative to the protein ([L]_T_/[P])^26^.

$${\mathrm{STD}}_{\mathrm{AMP}}=\frac{I_{0}-I_{\mathrm{STD}}}{I_{0}}\times \;[{L]}_{T}/[P]$$

As far as the group epitope mapping is concerned, these STD_AMP_ factors were then calibrated against the largest STD_AMP_ observed for each ligand, thus 100% corresponds to the signal with the largest STD effect.

#### 2.3.3. Competitive STD experiments

Competitive STD experiments with the established site markers, ibuprofen and warfarin were performed firstly on Bruker AV 500 MHz Spectrometer (Bruker Biospin, Rheinstetten, Germany). NMR samples were prepared in a 99.9% D_2_O buffer of 10 mM PBS, pH = 7.4. First, stock solutions of the ligands, rosmarinic acid, caffeic acid and salvianic acid and the site markers ibuprofen and warfarin were prepared in DMSO-*d*_6_*_._* The NMR samples were prepared in an NMR tube, of total volume 600 μL, containing 2 mM of the ligand and 50 μM of BSA, leading to a total ligand: protein ratio of 40:1. Then, titrations of ibuprofen at concentrations of 2 mM and 4 mM, respectively, took place. The total amount of DMSO-*d*_6_ in the NMR tube remained 10%. After each titration, the samples were subjected to STD experiment with a long protein saturation time of 2 s at 25 °C as described in paragraph 2.3.1. The same procedure took place with the Sudlow Site I site marker, warfarin. The only difference in this series of titrations was that the concentrations of warfarin were 2, 4, 6 and 8 mM, respectively, for each ligand–protein complex, due to better solubility.

Additional competition experiments with large excess of warfarin or ibuprofen were recorded on Agilent Technologies DD2 600 MΗz Spectrometer (NMR Centre, National Institute of Chemistry, Slovenia), using a cryoprobe, at 25 °C. The NMR samples were prepared in buffer 100% D_2_O, containing 10 mM Tris (98% *d*_11_). The pH was adjusted to 7.4, with the addition of DCl or NaOD. First, stock of the ligands (rosmarinic acid, caffeic acid, salvianic acid), as well as a stock of BSA solution, was prepared in Tris-*d*_11_ 10 mM buffer. On the contrary, stock of warfarin and ibuprofen were prepared in DMSO-*d*_6_. The samples were added in an NMR tube of total volume 600 μL. The concentrations of ligands were 0.2 mM and 20 μM of BSA, leading to a total ligand: protein ratio of 10:1. To each ligand–BSA complex 4 mM of warfarin was added. In the case of ibuprofen, the salvianic acid was added to the sample at 0.2, 0.4, 0.8, 1, 1.5, 2 and 3 mM concentration in 600 μL of total volume by keeping a constant concentration of ibuprofen at 2 mM. The concentration of BSA was 20 μΜ, leading to a ratio for the complex ibuprofen: BSA to 100:1. The total amount of DMSO-*d*_6_ in the tube remained 10%. The samples have been studied through STD NMR experiments. The experimental conditions which have been used for this series of experiments are the same with the conditions described in paragraph 2.3.2. The main difference is the long protein saturation time of 2 s which has been used.

#### 2.3.4. Tr-NOESY experiments

NOESY experiments were recorded using a Bruker AV 500 MHz spectrometer (Bruker Biospin, Rheinstetten, Germany), with the Topspin 2.1 suite. For NOESY experiments all the samples were prepared in a 99.9% D_2_O buffer of 10 mM PBS, pH = 7.4. First, stock solutions of the ligands, rosmarinic acid, caffeic acid and salvianic acid were prepared in DMSO-*d*_6_. The concentration of the ligands in the NMR tube (600 μL) was 2 mM. The NMR samples were subjected to NOESY experiments at 25 °C. NOESY experiments for 2 mM of each free ligand, rosmarinic acid, caffeic acid and salvianic acid, were recorded in two different mixing time values (d8): 0.1 s and 0.8 s, spectral width of 9.9773 Hz, 2048 data points, 56 number of scans, to obtain a spectrum where the NOE signals of the free ligand are well distinguished.

The additional tr-NOESY experiments were recorded on Agilent Technologies DD2 600 MHz and VNMRS 800 MHz Spectrometers (NMR Centre, National Institute of Chemistry, Slovenia), using a cryoprobe, at 25 °C. The stock solutions for the ligands rosmarinic acid, caffeic acid and salvianic acid, as well as a stock solution of BSA were prepared in buffer 100% D_2_O, containing 10 mM Tris (98% *d*_11_). The pH was adjusted to 7.4, with the addition of DCl or NaOD. The NMR samples were prepared in an NMR tube, of 600 μL total volume. The ligand concentration was 2 mM, while the protein concentration was 20 μM, leading to a total ligand: protein ratio of 100:1. The tr-NOESY spectrum of rosmarinic acid was acquired at 600 MHz with 8192 data points in t_2_, 128 complex points in t_1_, spectral width of 4807 Hz, 32 scans, mixing time of 350 ms and relaxation delay of 1.5 s. The tr-NOESY spectra of a 1:1 mixture of salvianic (2 mM) and caffeic acid (2 mM) in the presence of BSA (20 μΜ) were acquired at 800 MHz with 8192 data points on t_2_, 188 complex points in t_1_, spectral width of 8012 Hz, 64 scans, mixing time of 700 ms and a relaxation delay of 1.5 s. Spectra were zero-filled twice and apodized with a squared sine bell function shifted by π/2 in both dimensions.

### 2.4. Isothermal titration calorimety

The thermodynamic parameters of the interactions between BSA and rosmarinic, caffeic and salvianic acid were evaluated using the MicroCal ITC200 calorimeter and the results were processed through the Origin for Microcal ITC software. The values of the association constant (*K*_a_), the stoichiometry (*N*), the change of enthalpy (Δ*H*) and entropy (Δ*S*) were estimated directly from the software. The change of the Gibbs free energy (Δ*G*) was determined using the following equation:

ΔG° =−RTlnK

BSA, rosmarinic, caffeic and salvianic acid were dissolved in sodium phosphate buffer (0.1 M, pH = 7.4) and degassed thoroughly before loading. For each measurement BSA was loaded into the sample cell and the ligand (rosmarinic/caffeic/salvianic acid) was loaded into the syringe. The titrations were performed at 298 K. For the BSA–rosmarinic acid interaction, concentrations of 0.143 mM of BSA and 2.5 mΜ of rosmarinic acid were used, respectively. For the BSA–caffeic acid interaction, 0.148 mM of BSA and 2.5 mΜ of caffeic acid, were added, respectively. For the BSA–salvianic acid interaction 0.15 mM of BSA and 5 mΜ of salvianic acid were loaded, respectively, in the cell and the syringe. The three phenolic acids are soluble in the applied concentrations, and thus, the DMSO was avoided in contrast to the NMR experiments. The programmable titration was controlled by Origin for Microcal ITC software, and the titrant was injected into the sample cell in 20 portions. The first titration was of 1 μL volume and the subsequent titrations were of 2 μL each. Α continuous stirring of 1000 rpm was maintained during the titrations. The spacing between two injections was 200 s (after the first titration the spacing was 160 s) and the reference power was set to 6 μcal s^−1^. A 3 s filter period was applied. To correct the thermal effect due to mixing and dilution, control experiments were performed by injecting rosmarinic or caffeic or salvianic acid solution into buffer and by injecting buffer into BSA solution. The enthalpy change occurred by the buffer titrations to BSA was small but higher compared to the enthalpy change observed after titrating buffer into buffer, which may be due to the presence of dimers formed by BSA in the solution^29^. The emerging data were subtracted from the initial experiment and the thermodynamic values were calculated based on the one site binding model as it was the best-fitting model. For the interaction between BSA and caffeic acid the N value was set to 1.8 to improve the fitting.

### 2.5. Molecular docking

Bovine serum albumin (BSA) homodimer crystal structure was downloaded from the Protein Data Bank (https://www.rcsb.org/). The downloaded structure of BSA (PDB ID: 4F5S) constitutes the crystal structure of the apoenzyme – protein. BSA complexes with various bioactive molecules were not used in the study as the ligands deviated structurally with those under study. The homodimer protein was prepared using Maestro’s Protein Preparation Wizard. During this preparation, missing loops and side chains were fixed using Prime algorithm. Water molecules beyond 5 Å from het groups were deleted, disulphide bonds among Cys residues were created and hydrogens were added to the crystal structure. Overlapping atoms were minimised and different alternate positions of side chains were committed to a single one. Chain B of each homodimer was deleted to accelerate docking process. PROPKA was used to identify the protonation states of amino acids at neutral pH and OPLS3 force field was applied in order to minimise hydrogen atoms of the protein.

#### 2.5.1. Ligand preparation

We have used the five different ligands rosmarinic acid, caffeic acid, salvianic acid, warfarin and ibuprofen to be docked to the protein structures described above. All ligands’ 3 D structures were designed in Maestro’s 2 D sketcher with the appropriate chiralities and were minimised using Macromodel. The minimised structures were prepared using LigPrep. OPLS3 force field was used for geometry minimisation of the structures, while Epik was used for the protonation states of the ligands at neutral pH. The prepared structures by LigPrep were incorporated into Macromodel for generating conformations for each ligand. Macromodel uses OPLS3 force field and water as solvent in order to generate different conformations for each ligand and apply conformational search. During the conformational search, minimisation occurs, so as to reject the high energy conformations of the ligand using PRGC method.

#### 2.5.2. Induced fit docking

The molecular docking studies were performed using Maestro’s Induced Fit Docking method (Schrödinger, LLC, New York, USA). Ligands have been determined to be docked from a file containing all the possible conformations of each ligand generated by Macromodel. Protein preparation constrained refinement was not applied in the Glide docking stage, as we used the already prepared by Protein Preparation Wizard structure of the receptor. Trimming side chains automatically (based on B – factor) and Prime refinement of the protein side chains were applied and the docking process was accomplished by Glide/XP. Finally, the binding energy for both Sudlow’s sites and for each ligand was calculated.

## 3. Results

### 3.1. Synthesis of salvianic acid

**Figure 1.**
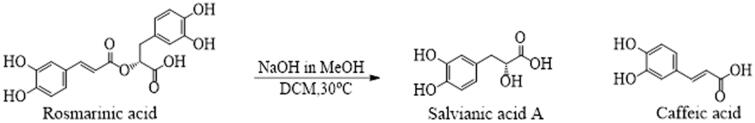
Hydrolysis of rosmarinic acid.

So far salvianic acid A was derived as an active ingredient of the plant *Salvia miltiorrhiza* where a massive quantity of plant material is used according to the patented procedure^30^ to yield 0.12–0.13% while other procedures result to even lower amount of salvianic acid^31^. Not only the isolation of salvianic acid A suffers low yield but, also, chemical synthesis reported from other research groups^32^^,^^33^ includes multistep reactions, low yield, several side-products and a considerable amount of chemical waste. In contrast, we present hereby a rapid, mild, one step methanolysis reaction utilising rosmarinic acid and disintegrate it to its precursors resulting to the two bioactive compounds, salvianic acid and caffeic acid, while any unreacted material is recovered and can be hydrolysed again. Various conditions were tested by altering the reaction time and the concentration of the alkaline solution, as it is presented in Table 1. The optimum reaction time is 4 h while in longer time, due to the alkaline conditions, a subsequent elimination is happening to salvianic acid resulting to caffeic acid (Figure 1).

**Table 1.** Reaction conditions tested to identify the optimum reaction conditions obtaining salvianic acid from rosmarinic acid.

Chromatogram (Supplementary Material Figure S1)	Conditions	Reaction time (h)	Salvianic acid yield (%)
**1**	Alkaline Methanolysis (with NaOH 0.1 M)	1	3
**2**	Alkaline Methanolysis (with NaOH 1 M)	4	22
**3**	Alkaline Methanolysis (with NaOH 1 M)	8	18
**4**	Alkaline Methanolysis (with NaOH 1 M)	12	12
**5**	Alkaline hydrolysis (with NaOH 1 M)	4	5
**6**	Alkaline hydrolysis (with NaOH 1 M)	8	2
**7**	Alkaline Methanolysis (with NaOH 0.1 M)	4	19

### 3.2. Interaction profile monitoring through fluorescence spectroscopy

**Figure 2.**
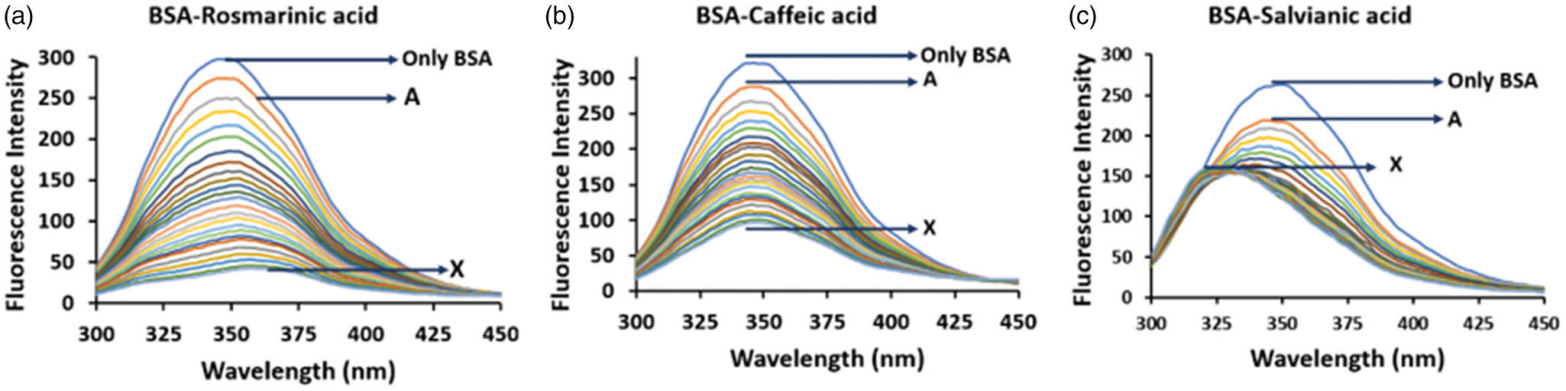
Fluorescence spectra of the interaction of BSA (2 μΜ) with (a) rosmarinic acid (A–Χ: 0–20 μΜ), (b) caffeic acid (A–Χ: 0–20 μΜ) and (c) salvianic acid (A–Χ: 0–20 μΜ).

The interaction of the three phenolic acids with BSA was first examined and confirmed through steady-state fluorescence spectroscopic studies by taking advantage of the intrinsic fluorescence of BSA. Upon excitation at 285 nm, BSA exhibits characteristic fluorescence signal which is mainly attributed in the fluorescence emitted by its tryptophan residues. However, the fluorescence intensity of the protein can be altered subsequently after the binding of a small molecule^23^^,^^24^^,^^34–36^. In Figure 2, the changes in the fluorescence spectrum of BSA are presented upon addition of rosmarinic, caffeic and salvianic acid. In all cases, the fluorescence intensity of the protein is decreased. In the cases of rosmarinic and caffeic acid the fluorescence quenching seems to follow the same pattern. In contrast, in the case of salvianic acid at low concentrations the fluorescence intensity decreases while at the same time a blue shift appears (∼ from 350 nm to 325 nm). As the concentration of salvianic acid rises the quenching rate is decreased reaching finally a plateau in the protein’s spectrum which according to the literature indicates. T hat the tryptophan residue(s) is located in a more hydrophobic environment^37^. This differential profile recorded among rosmarinic/caffeic acid and salvianic acid could be possibly rationalised due to different conformational changes occurring to the protein upon binding of the small molecules. Such irregular pattern has, also, been observed in the interaction of HSA with the terpenoid 16-*O*-methylcafestol and it was attributed to the conformational changes provoked by the ligand binding to Sudlow Site I as also to a fatty acid binding site^38^.

In addition, we estimated through the Stern–Volmer equation the quenching constant *K*_SV_ and the quenching rate constant *K*_q_ to compare the degree of the tryptophan fluorescence quenching of BSA in the presence of the three phenolic acids (Supplementary Material Section B, Figure S4 and Table S1). The order of the quenching efficiencies against BSA for the three phenolic acids is: rosmarinic acid > caffeic acid > salvianic acid with *K*_SV_ values equal to 42.4 × 10^4^, 17.1 × 10^4^ and 14.3 × 10^4^ L/mol, respectively.

### 3.3. Ligand epitope mapping through STD NMR

STD NMR can allow to screen the binding epitopes of ligands to proteins, as well as to determine the ligand protons that interact with the binding site of a protein or the active centre of an enzyme^3^^,^^36^^,^^38^^,^^39^. The strength of the interaction is also possible to be predicted, through the determination of the STD amplification factor. The STD NMR spectrum is recorded through subtraction of a spectrum in which saturation of the protein takes place. In the occurring difference spectra, the recorded signals report the protons of the ligand which interact with the protein^39^. A successful interaction between the ligand and the protein is apparent in the STD spectrum, through the appearance of low intense peaks related to the respective interacting protons.

**Figure 3.**
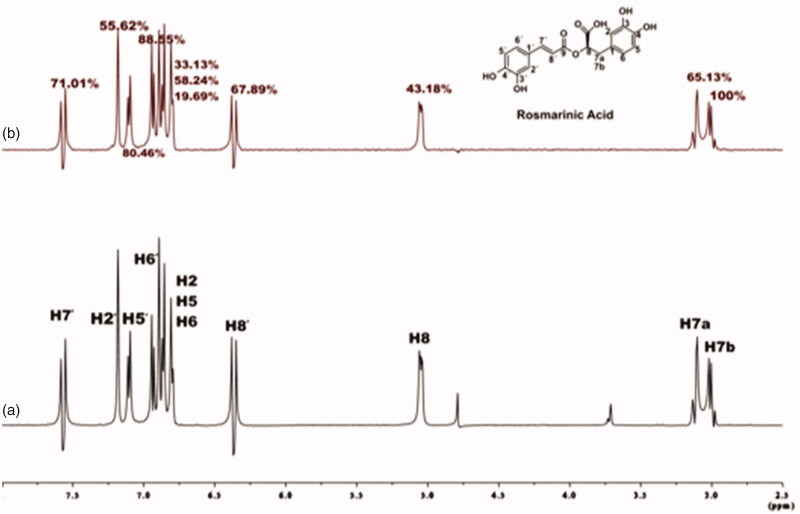
(a) ^1^H NMR reference spectrum of the complex rosmarinic acid (2 mM) – BSA (20 μM) in Tris-*d*_11_ buffer 10 mM, pH = 7.4 with 600 μL D_2_O. (b) STD difference NMR spectrum of the complex rosmarinic acid–BSA. The percentages values show the STD_AMP_ for all the protons of rosmarinic acid.

Of great concern was the determination of ligand ^1^H longitudinal relaxation time, T_1_, as it consists an important factor which can affect the intensities of STD signal^27^. We observed nonuniform relaxation properties across the molecule for all three studied ligands. The ligands’ ^1^H T_1_ values are in the range from 0.44 to 2 s. At long protein saturation time the differences in the T_1_ values are related to the differences in STD_AMP_ values, implying that the STD effect is directly affected by the ligand longitudinal relaxation time. In this case, an epitope mapping procedure for the ligand is inaccurate. In our work, we used both short and long protein saturation time. By using long saturation time of 2 s, leading to increased sensitivity of STD method, we confirmed the successful interaction of all the three ligands to the binding site of BSA (Figure 3; Supplementary Figures S5 and S6), whereas, by using short saturation time of 0.22 s, we the effect of longitudinal relaxation on STD effect and performed reliable epitope mapping for all the binding ligands to BSA (Figure 3; Supplementary Figures S5 and S6). The ligand: protein ratio was set to 100:1, to achieve better discrimination between strongly and weakly binding ligand moieties^26^.

For rosmarinic acid, the epitope mapping is illustrated in Figure 3. All the protons of rosmarinic acid (both the aromatic and the aliphatic) interact efficiently with BSA, showing STD_AMP_ values above 20%. The aromatic protons, H2΄, H5΄ and H6΄ interact with a STD_AMP_ values of 55.62%, 80.46% and 82.55%, respectively. The other group of aromatic protons H2, H5 and H6, show interaction with STD_AMP_ value of 33.13%, 58.24% and 19.69%, respectively. The vinylic protons H7΄ and H8΄, also bind sufficiently to BSA, with STD_AMP_ value of 71.01% and 67.89%, respectively. In addition, proton H8, which is placed in the α-position near the carboxylic acid group, shows satisfying interaction with a percentage of 43.18%. The last two signals in the STD spectrum refer to the two protons H7a and H7b, which belong to the β-CH_2_ group near the carboxylic acid moiety, and a strongest interaction to the binding pocket of BSA is observed for the H7b of the β-CH_2_ group near the carboxylic acid moiety, with STD_AMP_ value of 100%.

The epitope mapping of caffeic acid also showed interactions of all its protons with BSA (Section C1a; Supplementary Figure S5). Among all the protons, the strongest interactions are shown by the aromatic protons H9, H8 and H5, with STD_AMP_ values of 100%, 88.46% and 70.38%, respectively. The vinylic protons H2 and H3 show weaker interactions with the values of 56.79% and 66.01%, respectively.

Salvianic acid, the other substructural moiety of rosmarinic acid, also presents efficient STD effects of all protons (Supplementary Figure S6). The strongest effect is observed for the proton placed in the α-position near the carboxylic group, H2, with STD_AMP_ value of 100% (Section C1b). The aromatic protons, H8, H5, H9 bind efficiently to BSA, showing STD_AMP_ values of 28.07%, 57.89% and 31.58%, respectively. The proton H3a shows weaker interaction with BSA, with a value of 35.09%, as well as proton H3b which has STD_AMP_ value of 66.67%. As it is shown in Supplementary Figure S6, both stereoisomers of salvianic acid, which occur from the hydrolysis of rosmarinic acid, bind to BSA.

**Figure 4.**
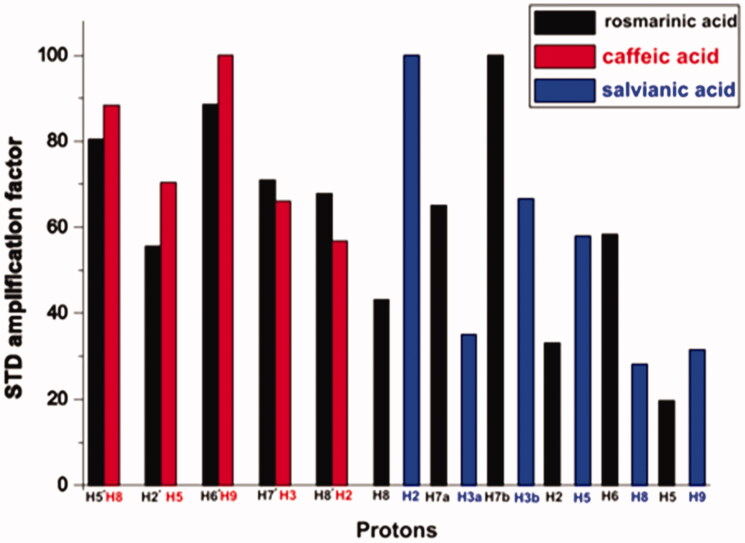
Relative STD_AMP_ (saturation transfer difference amplification factor) values of the saturated protons (regarding their binding to BSA) of rosmarinic acid (black) and its submoieties, caffeic acid (red) and salvianic acid (blue). The values in each molecule are normalised to the intensity of the signal with the largest STD effect.

The comparison of STD ligand epitope maps of the three BSA ligands is presented in Figure 4. Note that the relative values of the STD_AMP_ are normalised in each ligand separately. The strength of interactions with the protein can be compared only between protons inside a particular ligand. The intermolecular comparison is not appropriate because the magnitude of STD_AMP_ depends on the exchange kinetics of the ligand^26^. Nevertheless, differences or similarities in ligand binding profiles, important for the understanding of the ligand binding mode to specific protein target, can be extracted from such comparison plots^40^.

As it can be seen from the combined illustration of the STD epitope maps, there are similarities – in the appearance of STD maps of the same molecular scaffolds in the three ligands. Regarding to rosmarinic acid, the strongest STD effect appears to be in the aliphatic region of the molecule, and especially for proton H7b, followed by strong STD effect in the aromatic group of protons H6´ and H5´ with H5´ to present the strongest STD effect in the aromatic region.

For caffeic acid, the STD epitope map shows that the strongest STD effect is caused by the aromatic protons. What needs to be mentioned is that the strongest STD effect is caused by the aromatic protons H9 and H8 which are placed in the molecular structure at the same position as they are the aromatic protons H6´ and H5´ of rosmarinic acid. This leads us to the conclusion that this aromatic moiety in both ligands, interacts with BSA, in a similar way.

Also, salvianic acid, the other substructure of rosmarinic acid, shows some similarities in its STD epitope map with the corresponding moiety of rosmarinic acid. In both cases for these ligand moieties the strongest STD effect is observed in the aliphatic part. However, in salvianic acid, the strongest STD effect is caused by the aliphatic proton H2, which is in α-position near the carboxylic group. While, in rosmarinic acid, the strongest STD effect is caused by the aliphatic proton H7b, which is positioned near the second aromatic ring. This differentiation could be due to the neighbouring hydroxyl group that is present in salvianic acid.

### 3.4. Monitoring the conformation of rosmarinic acid and its substructures in the serum albumin bound state

In order to get a deeper insight on the BSA bound conformation of the three ligands, transferred NOE experiments were recorded. Tr-NOESY experiments can be a potent tool for determining the geometrical conformation that a ligand gains, when it interacts with the desired receptor^38^^,^^41^. Through this method, it can be confirmed if there is interaction between a ligand and a receptor at equilibrium and, thus, it can be widely used in drug design techniques for the determination of any conformational changes^42^.

**Figure 5.**
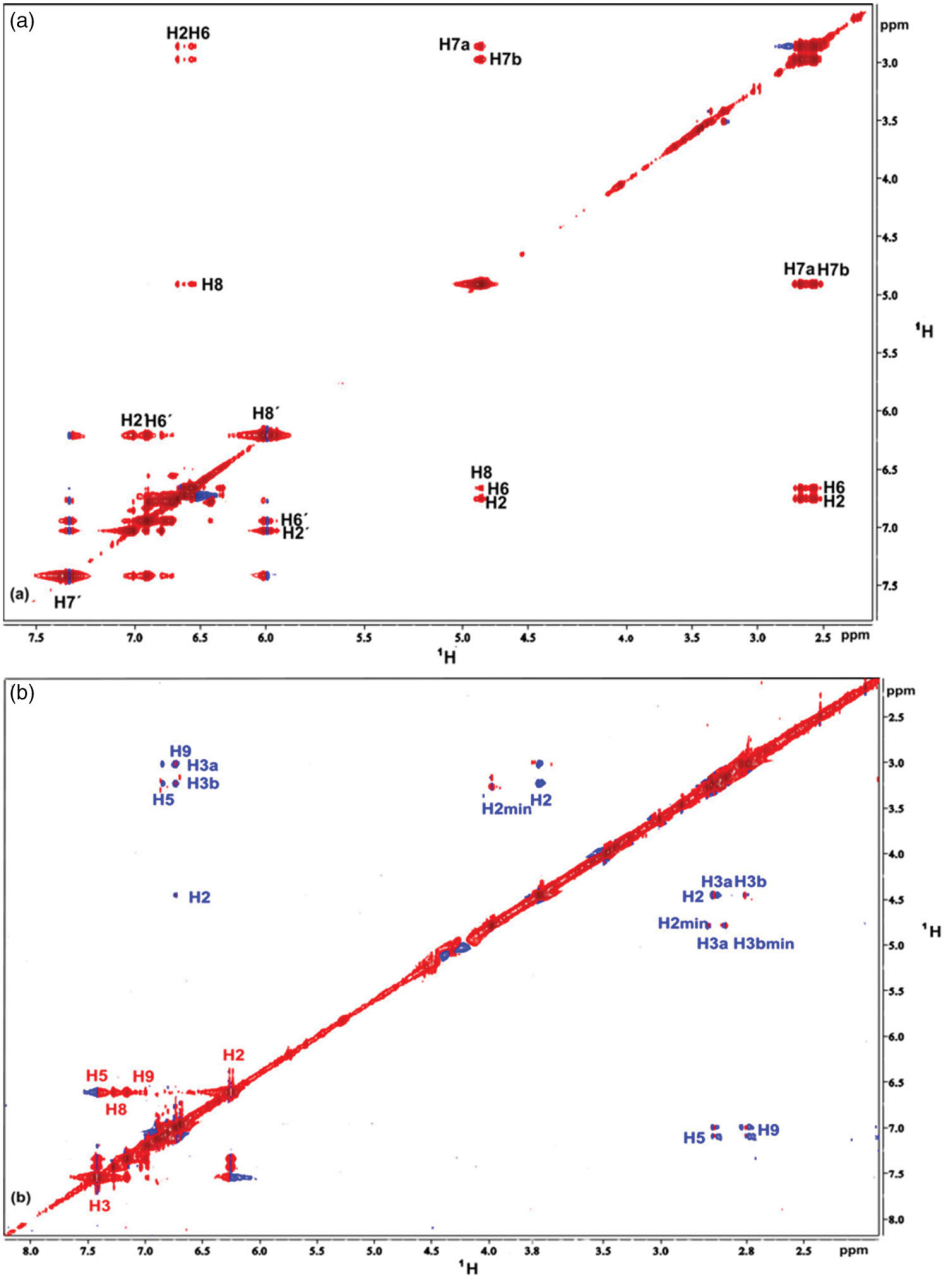
(a) Expanded region of tr-NOESY spectrum of the complex rosmarinic acid–BSA, recorded with mixing time of 350 ms at 600 MHz, showing the presence of negative NOEs of bound ligand. (b) Expanded region of tr-NOESY spectrum of the complex caffeic acid–salvianic acid–BSA, recorded with mixing time of 700 ms at 800 MHz showing the presence of negative NOEs, which are related to the protons of caffeic acid (labelled in red) and presence of positive NOEs, which are related to the protons of the major stereoisomer of salvianic acid (labelled in blue). For the minor isomer of salvianic acid only trivial NOEs between the H2 and H3 protons, which are overlaid with the zero-order artefacts of scalar coupling, are present.

NOESY experiments for rosmarinic, caffeic and salvianic acids were recorded in the absence and in the presence of the protein. In the absence of protein positive NOE signals were recorded, due to the fact that the free ligand is in the fast motion regime (Supplementary Figures S7, S9 and S11). After the addition of BSA in the solution, the NOE signals of all three ligands changed sign, gave negative NOE cross peaks, leading to the fact that there is a successful interaction between ligands and BSA (Supplementary Figures S8, S10 and S12). It has to be mentioned that, these tr-NOESY spectra were recorded with long mixing times to confirm binding to BSA. As a result, high spin diffusion occurred in the spectra. For this reason, additional tr-NOESY spectrum was recorded for rosmarinic acid with shorter mixing time, so as to properly inspect its bound conformation in the BSA binding (Figure 5(a)).

In the tr-NOESY spectrum that presents the interaction of rosmarinic acid with BSA only NOEs between proton pairs, which are close in space due to the molecular structure of rosmarinic acid itself, are observed. That means that only NOEs within the two subscaffolds arise. The extended conjugated system of rosmarinic acid hampers conformational rotation, thus, NOEs among the different subscaffolds are not observed. These observations lead us to the fact that when rosmarinic acid binds to BSA, it keeps its plain extended structure as observed for the free ligand in solution.

An additional tr-NOESY spectrum with long mixing time was recorded for a mixture of caffeic and salvianic acid (Figure 5(b)) to investigate the mutual location of the rosmarinic acid’s subscaffolds in the BSA binding site. As it is shown in Figure 5(b) (labelled in red), intense negative NOEs appear that are of the same sign as the diagonal peaks. These NOEs are related to the protons of caffeic acid and confirm its binding to the BSA also in the presence of salvianic acid. Conversely, positive NOEs (different sign of diagonal) are observed between the protons of salvianic acid (Figure 5(b), labelled in blue). Thus, the caffeic acid displaces salvianic acid, indicating that these two subscaffolds bind to the same binding pocket of BSA, as well as, that the caffeic acid is a stronger binder.

### 3.5. Determination of the BSA binding site for each ligand

As the successful interaction between rosmarinic acid, caffeic acid, salvianic acid and BSA, was confirmed, the next part of our work is focussed on the determination of the specific Sudlow Site, where each of the three ligands is binding stronger. For the confirmation of the interacted sites, competitive STD experiments were recorded, using warfarin and ibuprofen that are established site markers for Sudlow Site I and Sudlow Site II, respectively.

#### 3.5.1. Monitoring binding to Sudlow Site I of BSA with competitive STD NMR

Warfarin is an anticoagulant agent, and can be used as spy marker, due to its interaction with Sudlow Site I of BSA. The STD experiment for the complex warfarin–BSA reported that there is a strong interaction. All the aromatic protons of warfarin bind to Sudlow Site I of BSA with STD amplification values above 40% (Supplementary Figure S13). The –CH_3_ group of warfarin, as well as the α-H3 protons placed near the carboxylic group interact also with BSA.

**Figure 6.**
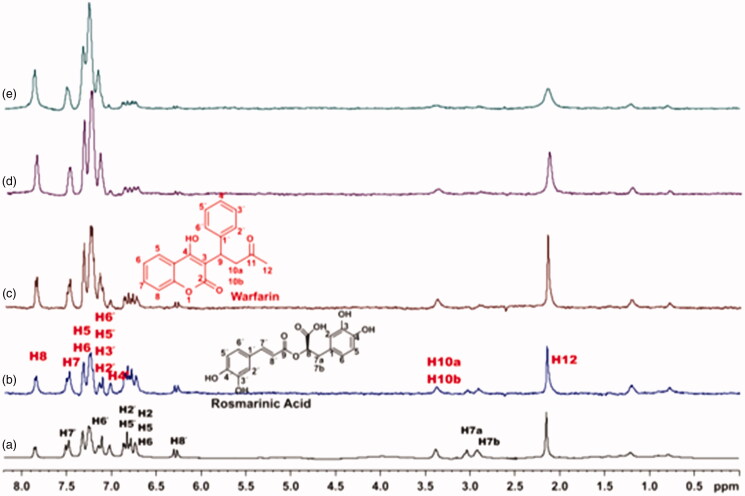
(a) ^1^H NMR reference spectrum of the complex rosmarinic acid (2 mM) – BSA (50 μΜ), including warfarin 2 mM, in PBS buffer 10 mM, pH = 7.4 with 600 μL D_2_O. STD difference NMR spectrum of the complex rosmarinic acid–BSA, including: (b) 2 mM warfarin. (c) 4 mM warfarin (d) 6 mM warfarin. (e) 8 mM warfarin (details for the protons of rosmarinic acid in Figure 3 and for warfarin in Supplementary Figure S13).

Competitive STD-NMR experiments for the complex rosmarinic acid–BSA, including warfarin were performed. The STD-NMR spectrum in Figure 6 reports that both ligands bind to Sudlow site I of BSA, as there is a reduction in the intensities of the peaks related to rosmarinic acid. Subsequently, titrations with warfarin at concentrations 4, 5 and 8 mM took place, so as to measure, through STD experiment, the concentration of the site marker needed for displacement of rosmarinic acid from Sudlow Site I (Figure 6(a–d)). As it is illustrated in Figure 6, when 8 mM of warfarin is added to the complex, the peaks related to rosmarinic acid in the STD spectrum, tend to be totally extinct. This leads to the fact that, warfarin almost completely displaces rosmarinic acid from BSA, at a concentration of 8 mM. The respective STD amplification factors, which are decreased as the concentration of warfarin upon titrations gets higher are shown in Supplementary Table S2.

**Figure 7.**
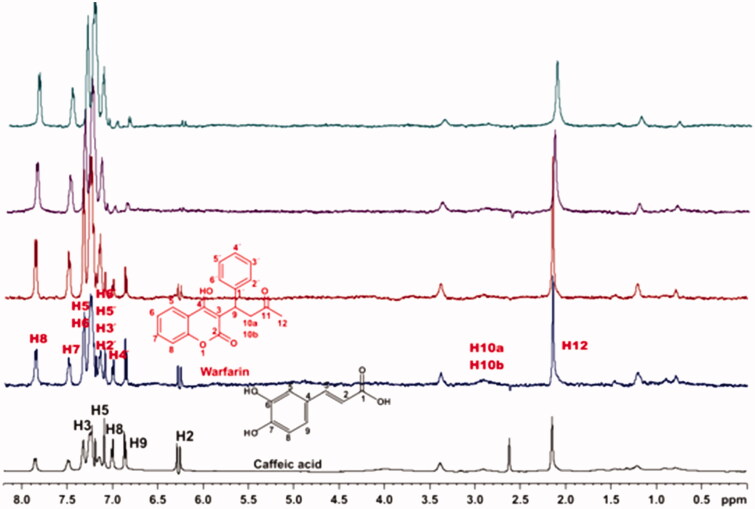
(a) ^1^H NMR reference spectrum of the complex caffeic acid (2 mM) – BSA (50 μΜ), including warfarin 2 mM, in PBS buffer 10 mM, pH = 7.4 with 600 μL D_2_O. STD difference NMR spectrum of the complex caffeic acid–BSA, including: (b) 2 mM warfarin, (c) 4 mM warfarin, (d) 6 mM warfarin, (e) 8 mM warfarin (details for the protons of caffeic acid in Supplementary Figure S5 and for warfarin in Supplementary Figure S13).

For caffeic acid, a similar procedure has been followed, as described above. Figure 7 shows the strong competition taking place between caffeic acid and warfarin, upon binding to Sudlow Site I of BSA. Titrations of warfarin to the complex of caffeic acid–BSA showed that 8 mM of warfarin can almost displace the caffeic acid from the Sudlow Site I. The STD amplification factors, which are decreased as the concentration of warfarin increases, are shown in Supplementary Table S4.

**Figure 8.**
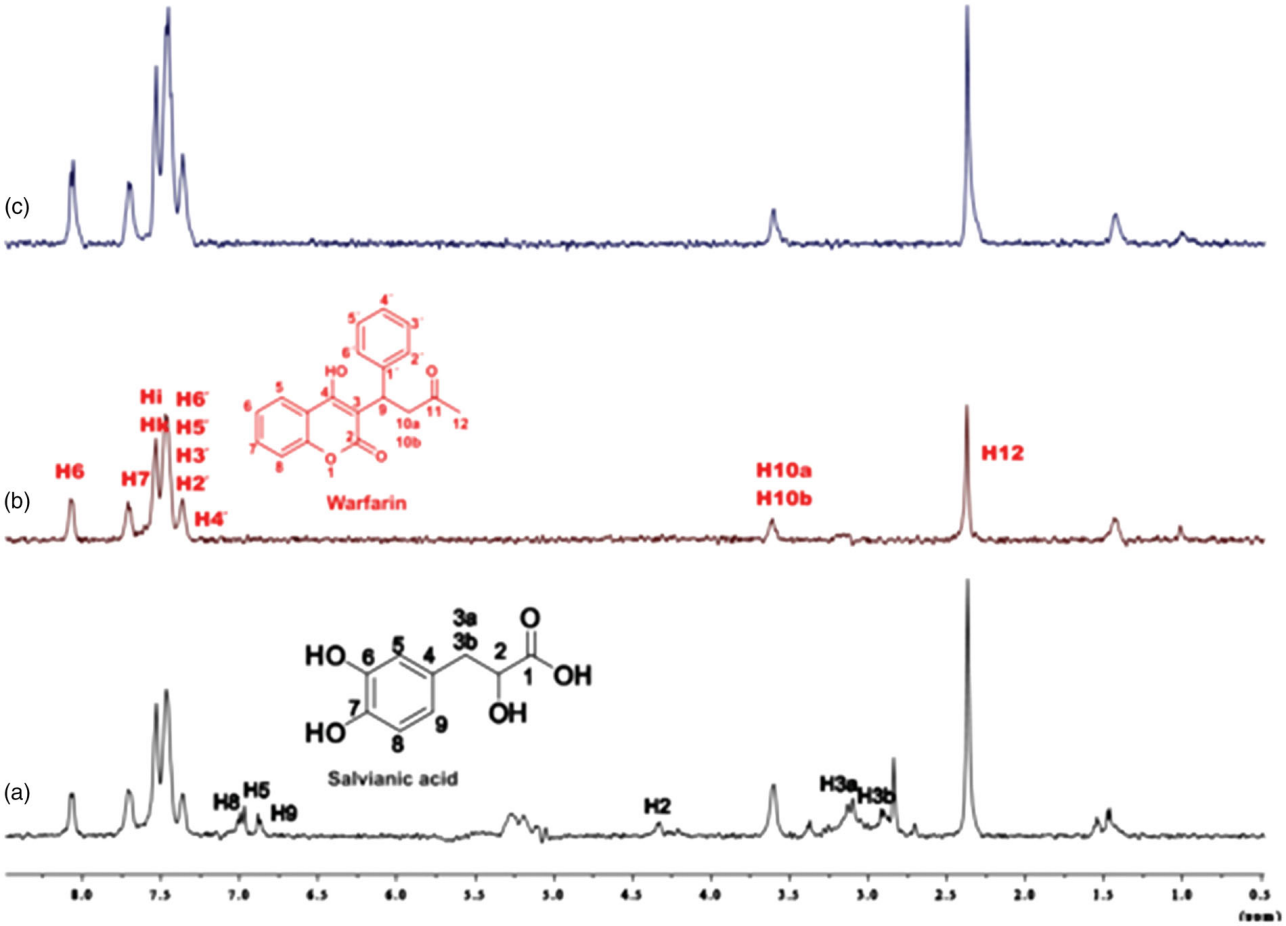
(a) ^1^H NMR reference spectrum of the complex salvianic acid (2 mM) – BSA (50 μΜ), including warfarin 2 mM, in PBS buffer 10 mM, pH = 7.4 with 600 μL D_2_O. STD difference NMR spectrum of the complex salvianic acid–BSA, including: (b) 2 mM warfarin, (c) 4 mM warfarin (details for the protons of salvianic acid in Supplementary Figure S6 and for warfarin in Supplementary Figure S13).

Competitive STD experiments for the complex of salvianic acid–BSA, including warfarin were also recorded (Figure 8). The STD-NMR spectrum of the complex salvianic acid–warfarin–BSA reported that the signals related to salvianic acid, disappear in the presence of isomolecular quantity of warfarin. So, as expected there is no signal of salvianic acid, when 4 mM of warfarin are added. This effect is in agreement with the fact that salvianic acid is a weak binder to BSA, and can be removed easily by a stronger binder, like warfarin, even in isomolecular concentrations.

**Figure 9.**
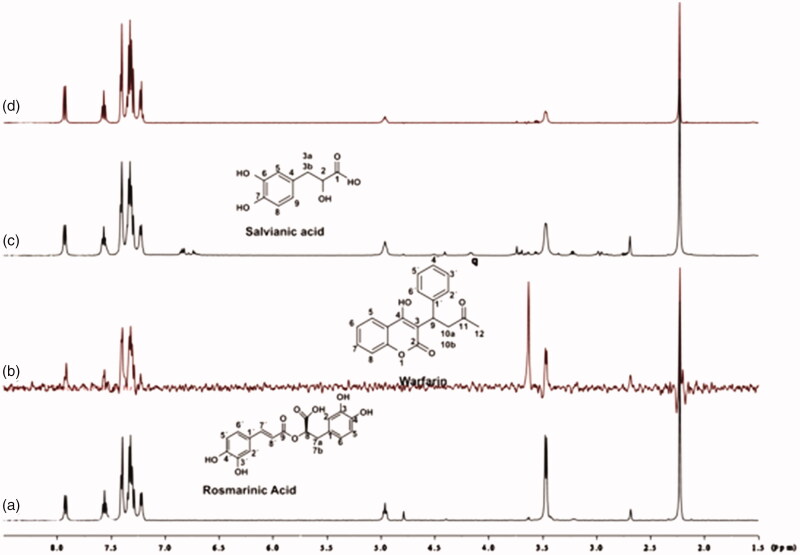
(a) ^1^H NMR reference spectrum of the complex rosmarinic acid–warfarin–BSA (0.2 mM:2 mM:20 μΜ), in Tris buffer 10 mM, pH = 7.4. (b) STD difference spectrum of the complex rosmarinic acid–warfarin–BSA (0.2 mM:2 mM:20 μΜ), in Tris buffer 10 mM, pH = 7.4. (c) ^1^H NMR reference spectrum of the complex salvianic acid–warfarin–BSA (0.2 mM:2 mM:20 μΜ), in Tris buffer 10 mM, pH = 7.4 (d) STD difference spectrum of the complex salvianic acid–warfarin–BSA (0.2 mM:2 mM:20 μΜ), in Tris buffer 10 mM, pH = 7.4 (details for the protons of rosmarinic acid in Figure 3, for salvianic acid in Supplementary Figure S6 and for warfarin in Supplementary Figure S13).

To confirm that there is a successful competition for the couples rosmarinic acid–warfarin, caffeic acid–warfarin and salvianic acid–warfarin, additional STD spectra were recorded. The difference in this case is that 4 mM of warfarin and only 0.2 mM of each rosmarinic and salvianic acid were used, towards, 20 μM of BSA. As it can be seen in Figure 9, in the STD spectra, there is no apparent signal related to the peaks of rosmarinic acid, caffeic acid and salvianic acid that would indicate binding to other BSA binding sites. This fact led us to the assumption that rosmarinic acid, caffeic acid and salvianic acid interact mainly with the Sudlow Site I.

#### 3.5.2. Monitoring binding to Sudlow Site II of BSA via competitive STD NMR

Ibuprofen belongs to non-steroids Pl family and is used as an anti-inflammatory agent. Due to its special binding to Sudlow Site II of BSA, it is easily used as spy marker in competitive experiments. As it is shown in the epitope map in Supplementary Figure S14, ibuprofen binds successfully to Sudlow Site II of BSA, with STD_AMP_ above 40%.

**Figure 10.**
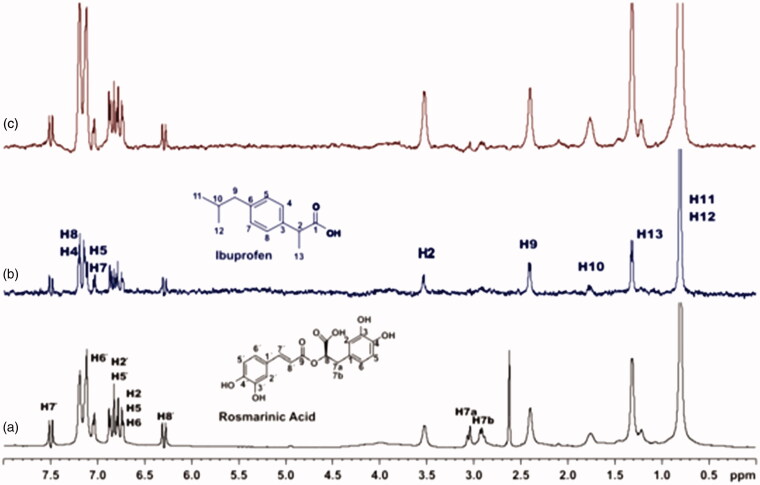
(a) ^1^H NMR reference spectrum of the complex rosmarinic acid (2 mM) BSA (50 μΜ), including ibuprofen 2 mM, in PBS buffer 10 mM, pH = 7.4 with 600 μL D_2_O. STD difference NMR spectrum of the complex rosmarinic acid–BSA, including: (b) 2 mM ibuprofen (c) 4 mM ibuprofen (details for the protons of rosmarinic acid in Figure 3 and for ibuprofen in Supplementary Figure S14).

**Figure 11.**
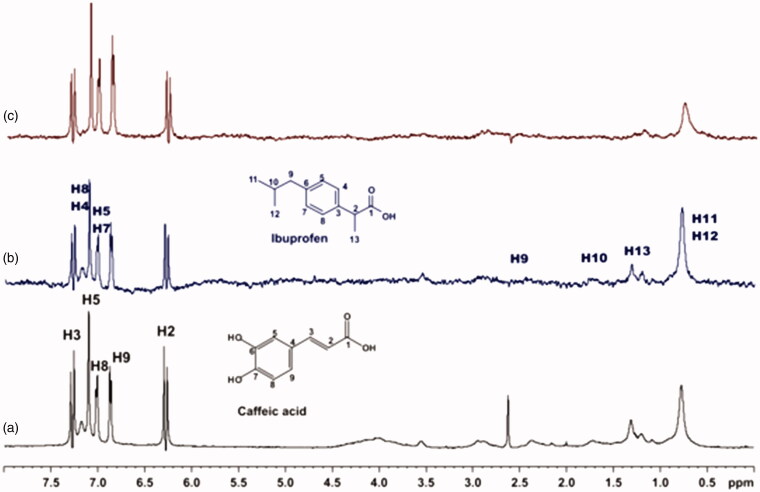
(a) ^1^H NMR reference spectrum of the complex caffeic acid (2 mM) – BSA (50 μΜ), including ibuprofen 2 mM, in PBS buffer 10 mM, pH = 7.4 with 600 μL D_2_O. STD difference NMR spectrum of the complex caffeic acid–BSA, including: (b) 2 mM ibuprofen (c) 4 mM ibuprofen (details for the protons of caffeic acid in Supplementary Figure S5 and for ibuprofen in Supplementary Figure S14).

**Figure 12.**
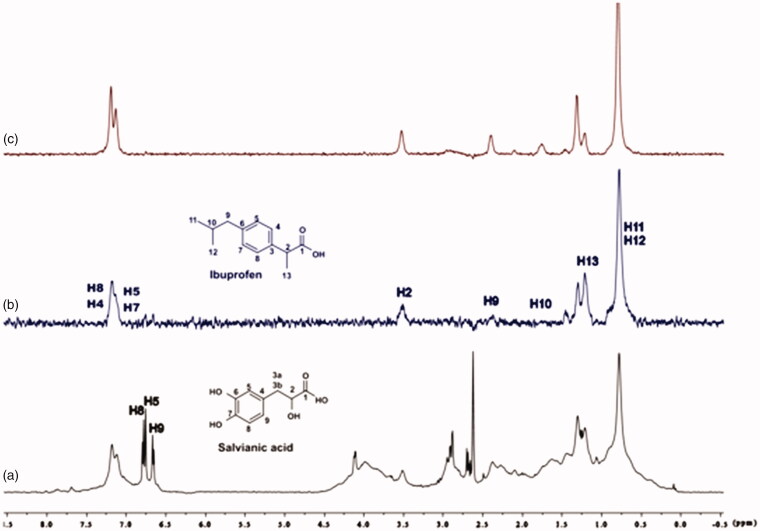
(a) ^1^H NMR reference spectrum of the complex salvianic acid (2 mM) – BSA (50 μΜ), including ibuprofen 2 mM, in PBS buffer 10 mM, pH = 7.4 with 600 μL D_2_O. STD difference NMR spectrum of the complex salvianic acid–BSA, including: (b) 2 mM ibuprofen, (c) 4 mM ibuprofen (details for the protons of salvianic acid in Supplementary Figure S6 and for ibuprofen in Supplementary Figure S14).

**Figure 13.**
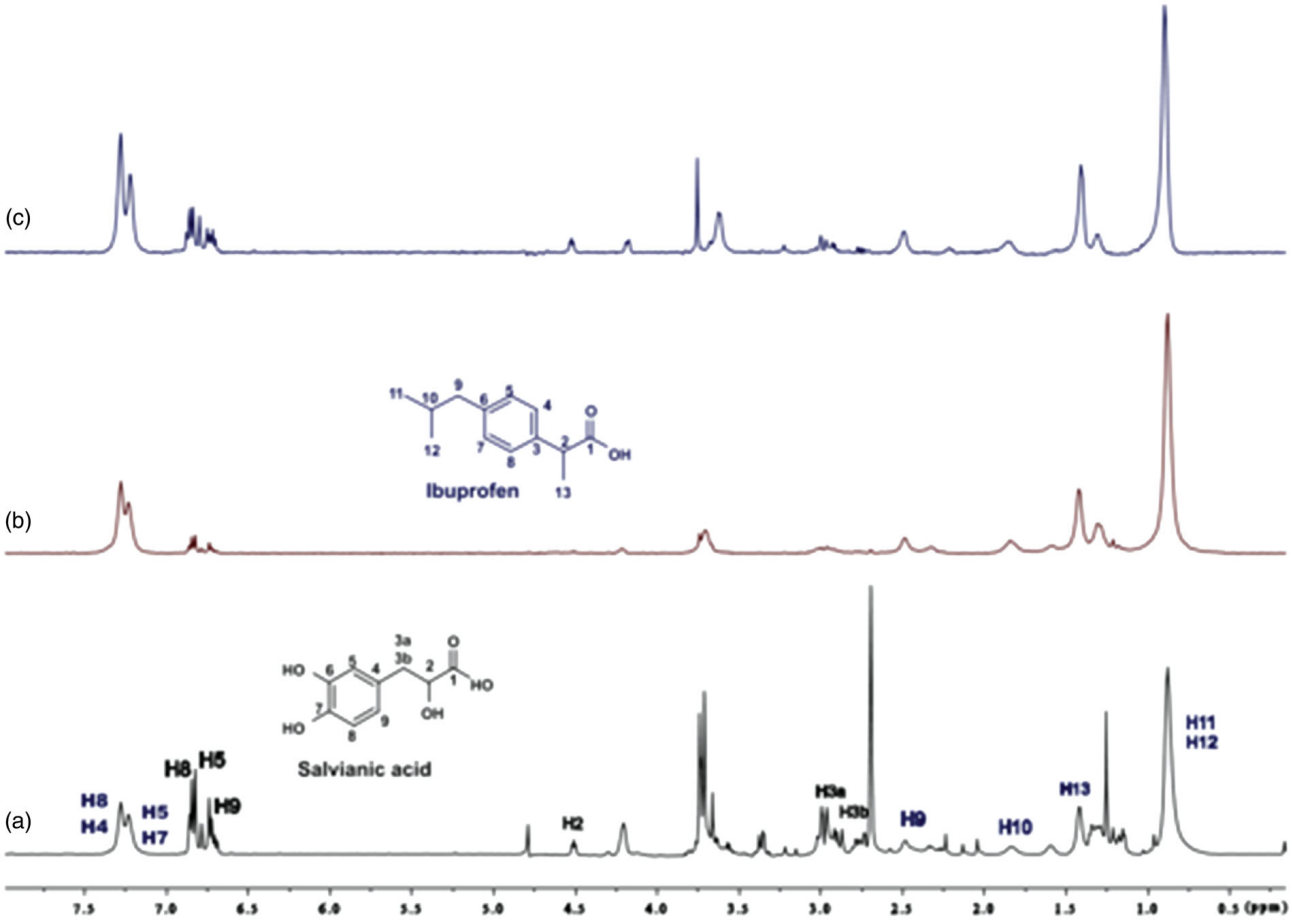
(a) ^1^H NMR reference spectrum of the complex ibuprofen (2 mM) BSA (20 μΜ), including salvianic acid 0.2 mM, in Tris-*d*_11_ buffer 10 mM, pH = 7.4 with 600 μL D_2_O. STD difference NMR spectrum of the complex ibuprofen–BSA, including: (b) 0.2 mM salvianic acid, (c) 3 mM salvianic acid (details for the protons of salvianic acid in Supplementary Figure S6 and for ibuprofen in Supplementary Figure S14).

For the titrations of each complex ligand: BSA with ibuprofen, we followed the same procedure as with warfarin. The main difference for this series of experiments, was that the total concentration of the ibuprofen titrated was 4 mM, because of the low solubility of ibuprofen in the buffer. Although, 4 mM of ibuprofen towards 2 mM of each ligand (ratio 2:1), were enough to show that the presence of ibuprofen does not affect the binding of each ligand to BSA, as their signals are observed in the STD spectra (Figures 10–12). An exception could stand for salvianic acid, in which the titration of 2 mM of ibuprofen, seemed to displace salvianic acid in the respective STD spectrum (Figure 12). However, additional titration experiments were conducted (Figure 13; Supplementary Figure S15) by keeping a constant concentration of ibuprofen at 2 mM and adding salvianic acid. These competitive STD experiments showed that salvianic acid is not competing with ibuprofen at the Sudlow Site II, as the STD_AMP_ of ibuprofen is constant at different concentrations of salvianic acid (See Graph S1 in Supplementary Material), although, the STD_AMP_ value of the protons of salvianic acid is increasing (Supplementary Table S8). Moreover, according to the Supplementary Tables S3, S5 and S7, there is an increase in the values of STD_AMP_ as the concentration of ibuprofen is increasing. This fact led us to the conclusion that ibuprofen could accelerate the binding of each ligand (rosmarinic, caffeic and salvianic acid) to Sudlow Site I pocket of BSA, due to the presence of allosteric effect.

### 3.6. Revealing the thermodynamics of the BSA–ligand interactions with ITC

**Figure 14.**
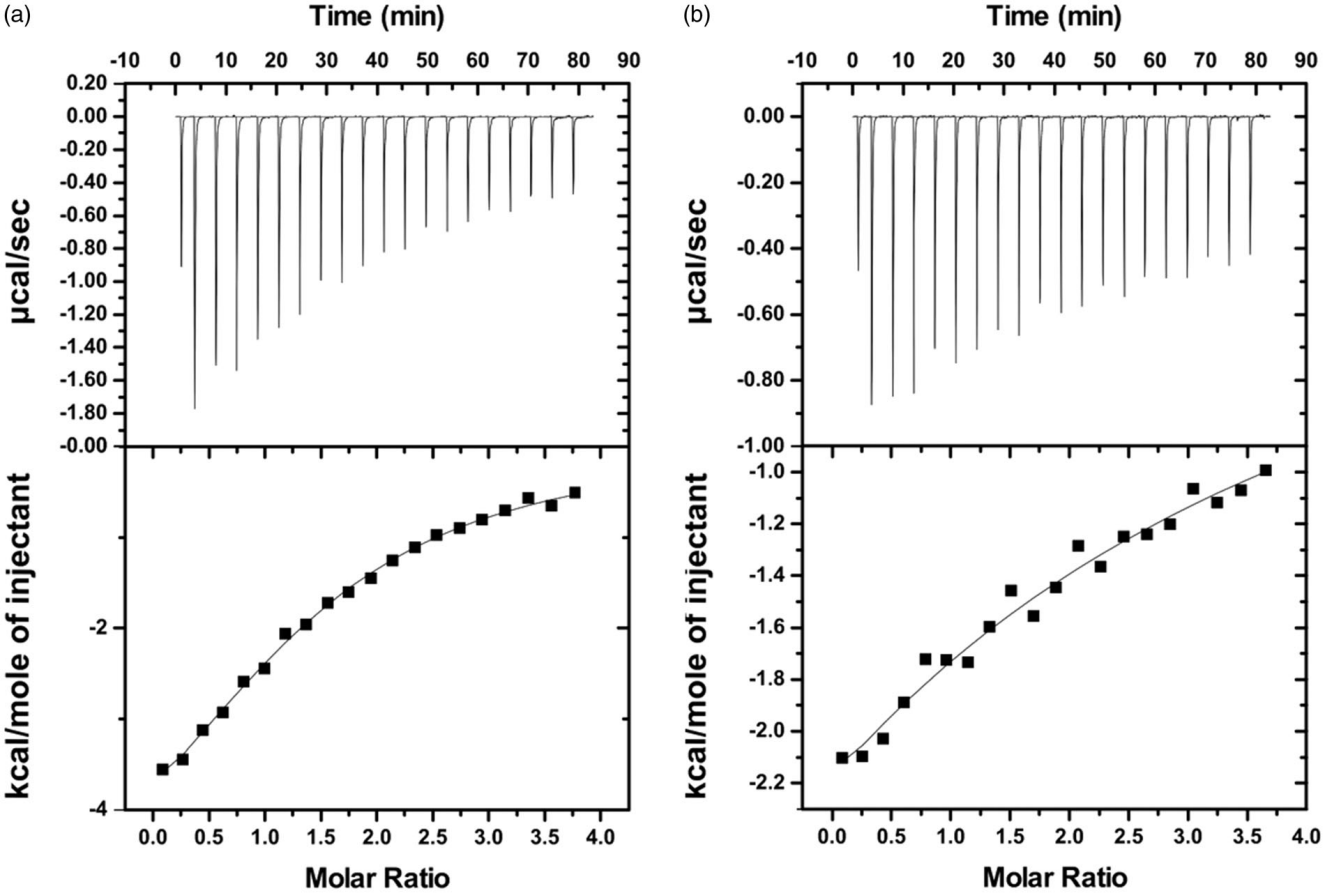
Isothermal titration calorimetry measurements for the interaction of BSA with (a) Rosmarinic acid and (b) Caffeic acid. For each interaction the isotherm plot (up) and the fitting of the integrated curve (down) are being presented.

ITC measurements were employed to evaluate the thermodynamic parameters and the binding affinity between BSA and the three phenolic acids (Figure 14). Interestingly, the results displayed significant differences between the binding affinities of the molecules with BSA. The low affinity of salvianic acid to BSA did not allow the estimation of the thermodynamic parameters characterising their interaction (Supplementary Figure S16). However, a moderate affinity to BSA was determined (Table 2) for the BSA–rosmarinic acid interaction (*K*_d_=135.3 ± 10.8 μΜ) and a weak affinity for the BSA–caffeic acid interaction (*K*_d_=1564 ± 156.7 μΜ). Hence, the ranking order of binding of the different molecules (from higher to lower affinity) for BSA is the following: rosmarinic acid > caffeic acid > salvianic acid.

**Table 2.** Thermodynamic parameters and binding affinities of the BSA interaction with rosmarinic and caffeic acid.

Ligand	*n*	Δ*Η* (cal/mol)	Δ*G* (cal/mol)	Δ*S* (cal/mol/deg)		*K*_a_ (M)	*K*_d_ (μΜ)
Rosmarinic acid	1.45	−6008 ± 302.8	−5280	−2.44	7390 ± 593		135.3 ± 10.8
Caffeic acid	1.8	−14680 ± 1139	−3832	−36.4	639 ± 65.3		1564.9 ± 156.7

Regarding the thermodynamics, the interactions of BSA with rosmarinic acid and caffeic acid are exothermic and spontaneous since the changes in enthalpy (Δ*H* < 0) and Gibbs free energy are negative (Δ*G* < 0). Given that in both cases the change in entropy is, also, negative, we can conclude that the association is enthalpically driven, and thus, mainly dominated by hydrogen bonding and electrostatic interactions.

### 3.7. The structural architecture of the interactions with serum albumin using molecular docking

To provide details in the important molecular interactions of the various molecules studied with the Sudlow site I of BSA and potentially explain their competitive properties, molecular docking studies were performed. The docking scores are diagnostic of the molecules ability to interact at the binding site, and therefore, to give some explanation on the competitive NMR results.

**Figure 15.**
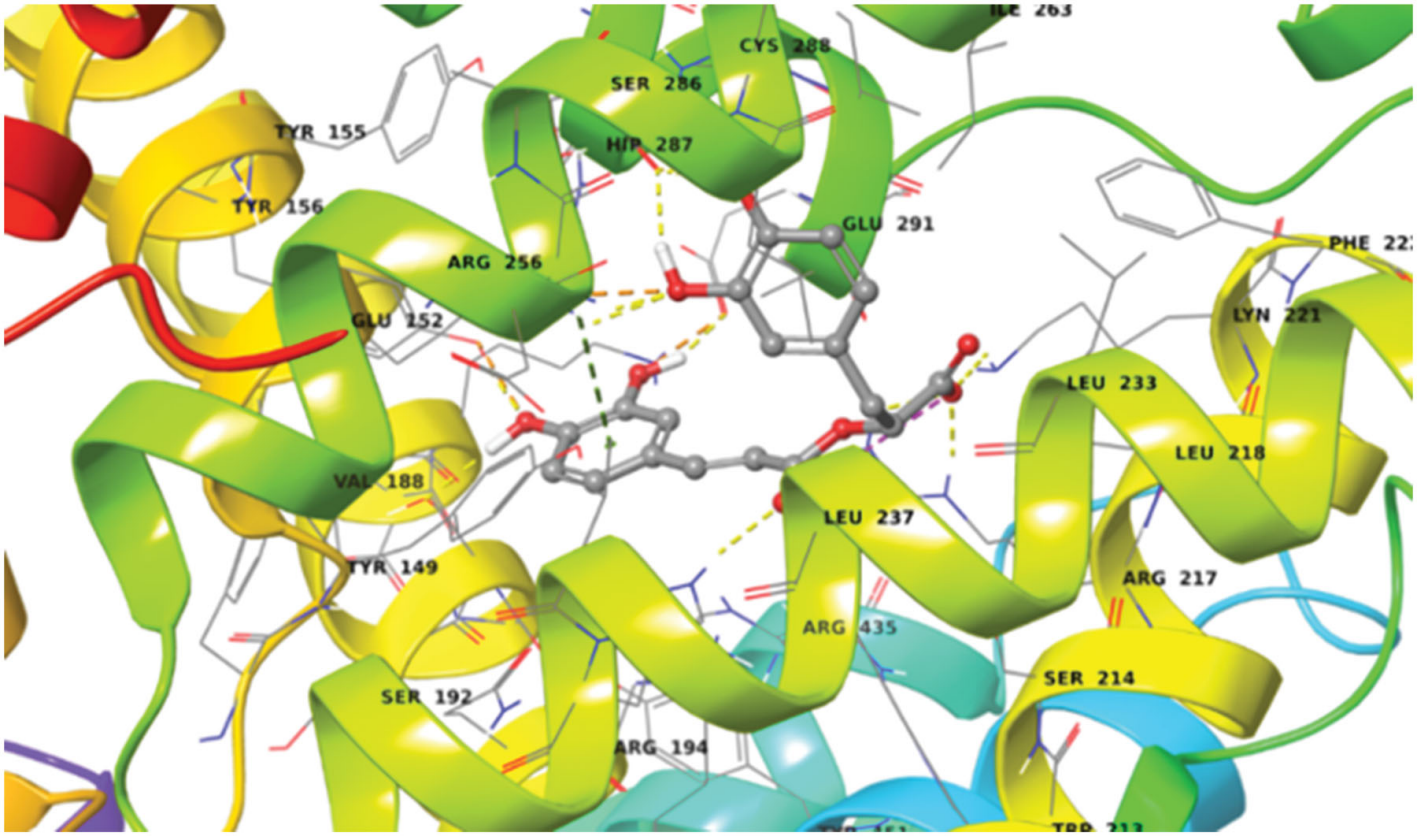
Best pose of rosmarinic acid in Sudlow site I. The nine favourable hydrogen bonds and π-π stacking between Arg256 and aromatic ring A (Supplementary Figure S17) can explain its highly favourable binding.

The first ligand docked to Sudlow site I of BSA is rosmarinic acid. This exhibited a very favoured binding value (Docking Score = −13.545 kcal/mol) in Sudlow site I (Supplementary Table S8). Rosmarinic acid in its best pose conformation (Figure 15; Supplementary Figure S17) forms nine H-bonds with eight residues of the protein (Tyr156, Ser191, Glu291, Arg256, Ser286, Lys221, Arg217, His241). In addition π-π stacking interaction between the aromatic ring A of the ligand and Arg256 plays a key – role in the stabilisation of the complex.

**Figure 16.**
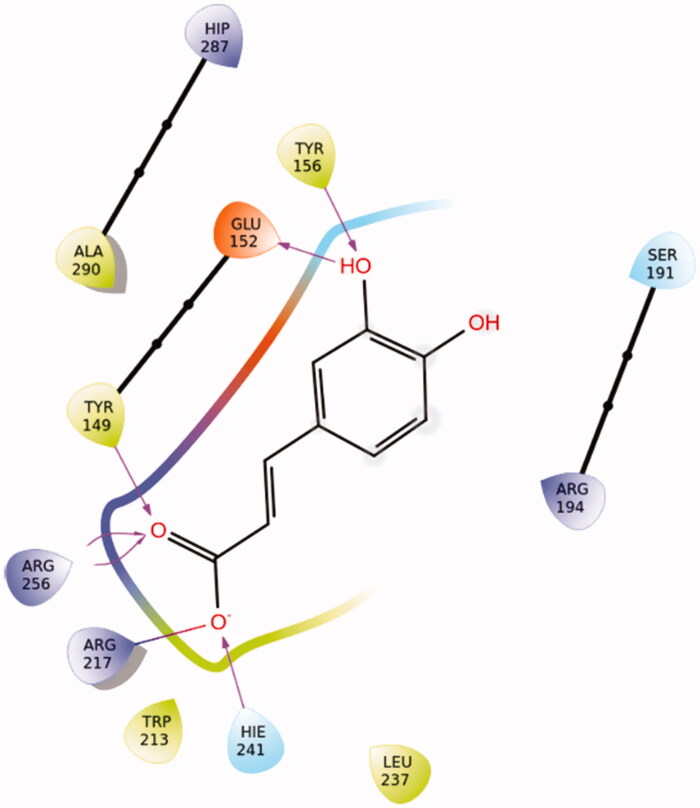
Binding interactions of caffeic acid in Sudlow site I of BSA.

Caffeic acid showed a very favoured binding value (Docking Score = −11.583 kcal/mol) in Sudlow site I (Supplementary Table S9) in accordance with STD experiments. Caffeic acid in its best pose conformation (Figure 16) forms H-bonds with five residues of the protein (Tyr156, Glu152, Tyr149, Arg256, His241). In addition, the formation of a salt bridge between Arg217 and the carboxylic group of the ligand contributes to the stabilisation of the molecule in the binding site.

**Figure 17.**
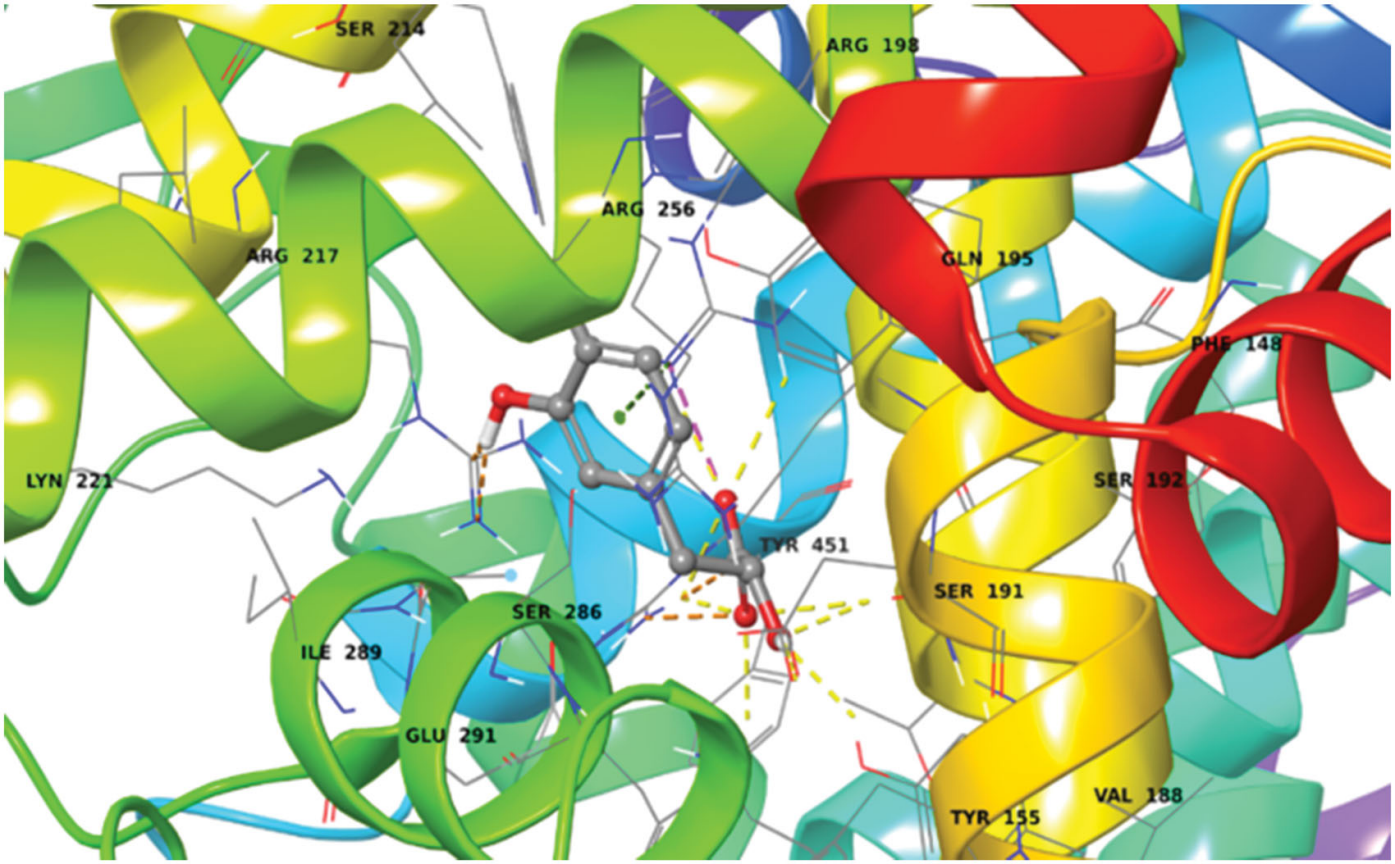
Best pose of BSA–salvianic acid in Sudlow site I. The four favourable hydrogen bonds, the salt bridge between Arg198–carboxylic anion and the π–cation can explain its highly favourable binding.

Salvianic acid shows also a very favoured binding value (Docking Score = −9.849 kcal/mol) in Sudlow site I (Supplementary Table S9). In its best pose conformation (Figure 17; Supplementary Figure S18) forms H-bonds with four residues of the protein (His241, Arg194, Lys187, Glu152). In addition, the formation of a salt bridge between Arg198 and the carboxylic group of the ligand and the stabilisation of the π-cation by Arg198 contribute to the stabilisation of the molecule in the binding site.

In Table 3, hydrogen distances of all the H atoms presenting NOE signals (2 D NOESY spectra of Figure 5) are measured. According to these measurements, the conformations of all three molecules predicted by molecular docking studies (Figures 15–17) are compatible with those observed with tNOEs.

**Table 3.** Hydrogens’ distances (presenting tr-NOE signals) in their best pose conformations in Sudlow’s I cavity.

Molecule in Sudlow’s I cavity	Hydrogens presenting tr-NOE signals		Strength of NOEs	Hydrogen distances (Å)
Rosmarinic acid	H_6_	H_7a_	s	2.45
	H_6_	H_7b_	m	3.63
	H_2_	H_7a_	m	3.72
	H_2_	H_7b_	s	2.53
	H_8_	H_6_	w	4.44
	H_8_	H_2_	w	4.10
	H_8’_	H_2’_	s	2.24
	H_8’_	H_6’_	w	4.60
Caffeic acid	H_3_	H_5_	s	2.63
	H_3_	H_9_	m	3.83
Salvianic acid	H_5_	H_3a_	m	3.56
	H_5_	H_3b_	s	2.40
	H_9_	H_3a_	s	2.60
	H_9_	H_3b_	m	3.75

## 4. Discussion

Rosmarinic acid consists of two phenolic acids, caffeic and salvianic acid. The enzymatic formation of caffeic and salvianic acid arise from 4-coumaroyl-CoA and 4-hydroxyphenyllactic acid, respectively, which are, also, the two basic biosynthetic precursors of rosmarinic acid^43^^,^^44^. Thus, plant biosynthetic machineries have evolved to combine simpler structural components to generate molecules with higher structural complexity. Whether this structural complexity leads to greater biological potency depends on the specific target. For example, the binding of rosmarinic acid to BSA reduces its inhibitory effect against acetylcholinesterase and at the same time enhances its antioxidant capacity^45^.

Rosmarinic and caffeic acid have been previously studied on their interaction with serum albumin (BSA or HSA)^9^^,^^46–48^ while there is only one study for the interaction of salvianic acid with BSA^49^. Fluorescence spectroscopy is the dominant technique used to elucidate these interactions. However, here for the first time the binding of rosmarinic acid to BSA is investigated in relation to the binding of its two submoieties to the same protein in a more detailed way by utilising sophisticated techniques. In addition, we describe a synthetic process with improved yield for salvianic acid.

Salvianic acid, a natural product originally isolated from the plant *Salvia miltiorrhiza,* is a bioactive compound presenting antioxidant and anti-inflammatory properties^50^^,^^51^. *In vivo*, it has illustrated cardioprotective effects in rats with myocardial infraction^52^ and myocardial ischaemia injury^53^, and mice with increased levels of homocysteine^51^ while it, also exhibited protective effects in mice with acute liver injury^54^. The above evidence indicates the therapeutic potential of salvianic acid and further investigation in preclinical and clinical stages could reveal its benefits in human health. However, currently large clinical trials or large scale studies are limited due to the restricted availability of salvianic acid. This is a common problem for natural products^55^, as only small amounts can be isolated. In such cases, total organic synthesis can assist. Many synthetic procedures for salvianic acid have been reported including its synthesis from commercially available compounds such as 3,4 dihydroxy benzaldehyde^15^^,^^16^^,^^19^ and its hydrolysis from rosmarinic acid and other natural products with both chemical and enzymatical reactions^17^^,^^20^^,^^56–58^. Although, these methods suffer from low yields, many reaction steps that diminish the yield and also produce a large amount of waste. To overcome these drawbacks, we applied the methanolysis of rosmarinic acid to its precursors with mild conditions and no waste production. To achieve that, different conditions were tested ending up to an optimum 4 h reaction utilising 1 M NaOH, while the unreacted material are the bioactive compounds caffeic and rosmarinic acids which can be further hydrolysed. We, also, observed that longer reaction times led to larger amounts of caffeic acid while the starting material was completely consumed and almost no salvianic acid was recovered. The main reason for this is the subsequent elimination of the produced salvianic acid due to the alkaline conditions as reaction time progresses.

The interaction with BSA of all three phenolic acids was first confirmed through fluorescence spectroscopy. The three phenolic acids caused quenching of the BSA’s tryptophan fluorescence, with rosmarinic and caffeic acid following a similar trend. By contrast, the titration of salvianic acid raised a large blue shift suggesting alterations in the protein conformation. As it was aforementioned, a similar quenching pattern was observed for an HSA–terpenoid interaction where the authors support that the ligand binding affects not only Sudlow Site I but also a fatty acid binding site^38^. Here, the NMR competitive experiments showed that salvianic acid binds to Sudlow Site I while at the same time its affinity with BSA was too low to be measured by ITC. However, the potential that salvianic acid could interact with a much weaker affinity to a fatty acid binding site could not be excluded. The binding interactions were further enlightened with STD NMR, a useful tool for the determination of the interaction between a protein or an enzyme and a ligand. Herein, we used this method for mapping of the interacting epitopes of each of the three ligands with BSA. To our knowledge, no STD NMR experiments have been reported for rosmarinic and salvianic acid^59^. The simultaneous evaluation of the interaction profile of rosmarinic acid and its constituents could provide a global view on the driving forces that each component contributes to the final interaction on the integral compound. The STD_AMP_ factors showed that the molecular structure of each ligand affects the epitope mapping of each proton. Their preference for Sudlow site I as it was derived from the competitive STD NMR experiments confirms the existing literature data^46^^,^^49^^,^^59^.

Concerning the affinities of the three molecules to BSA as they were estimated from the ITC experiments, the one of rosmarinic acid was unequivocally stronger. The BSA–caffeic acid interaction exhibited a weak affinity while the BSA–salvianic acid interaction was too low to be measured. This result is corroborated by the tr-NOESY competitive experiment (Figure 5(b)) where the addition of caffeic acid caused the displacement of salvianic acid from the protein’s cavity, implying that there is sufficient competition between these two molecules for the same binding pocket, with caffeic acid interacting more strongly than salvianic acid. It must be mentioned that the reported *K*_a_ values for the BSA–rosmarinic/caffeic acid interactions are higher and in the range of 10^4^–10^5^ M^−1^ ^46^^,^^60–63^. This discrepancy may be due to the different assay applied for the *K*_a_ determination. In all cases, the *K*_a_ was estimated through fluorescence spectroscopy indirectly as the technique measures the changes of the tryptophan fluorescence upon ligand binding. In contrast, ITC measures directly the heat of an interaction. Furthermore, high concentrations of the interactants (in the grade of mM for rosmarinic and caffeic acid) needed to be used for the ITC experiments in order to detect the binding. Generally, ITC is very sensitive, and thus, requires low concentrations for high affinity interactions. The BSA–rosmarinic/caffeic acid interactions were found to be enthalpically driven and possible H-bond formations are presented by the molecular docking studies. The best fitting model was the one binding site model supporting the STD NMR competitive results. Finally, a *K*_a_ = 1.17 × 10^5^ M^−1^ is reported for the BSA–salvianic interaction^49^ again estimated with fluorescence spectroscopy. In our experiments the binding was too low to estimate the *K*_a_ or the thermodynamic parameters using ITC. Besides, our finding that caffeic acid replaces salvianic acid in BSA opposes the reported affinity.

## 5. Conclusion

In conclusion, we presented a synthetic procedure to produce salvianic acid. The reaction in comparison to previous attempts reported has improved yield and no waste products. Additionally, an array of different biophysical techniques including fluorescence spectroscopy, ligand-based NMR screening and calorimetry methods as well as molecular docking were recruited for the analysis of the binding properties of rosmarinic acid and its two structural constituents caffeic and salvianic acid with BSA. All ligands were proved to bind to Sudlow Site I of BSA while the protein exhibits stronger affinity to rosmarinic acid followed by caffeic acid and eventually by salvianic acid. The strongest binding of rosmarinic acid to BSA can be accredited to its structural complexity rather than to the binding properties of its two isolated substructures. Thus, through evolution, nature has reused these two building blocks to build up new chemical space and we show that this chemical space delivers a more favourable interaction profile with proteins and specifically with serum albumin.

## Supplementary Material

Supplemental Material
