# Supplementary material for: Unveiling the interaction profile of rosmarinic acid and its bioactive substructures with serum albumin
Source: J Enzyme Inhib Med Chem. 2020 Mar 23;35(1):786–804. doi: 10.1080/14756366.2020.1740923 (PMC7144280; doi:10.1080/14756366.2020.1740923)
Supplement: Supplemental Material [file IENZ_A_1740923_SM9522.pdf]

Supporting Information

Unveiling the interaction profile of rosmarinic acid and its bioactive substructures with serum albumin

Christina Papaemmanouil[1], Maria V. Chatziathanasiadou[1], Christos Chatzigiannis[1], Eleni Chontzopoulou[2], Thomas Mavromoustakos[2], Simona Golic Grdadolnik[3], Andreas G. Tzakos[1]

[1]Department of Chemistry, Section of Organic Chemistry and Biochemistry, University of Ioannina, Ioannina 45110, Greece

[2]Department of Chemistry, National and Kapodistrian University of Athens, Panepistimiopolis Zografou 15771, Greece

[3]Laboratory of Biomolecular Structure, Theory Department, National Institute of Chemistry, Hajdrihova 19, 1001 Ljubljana, Slovenia

Table of Contents

A. Characterization of salvianic acid 32
A1. HPLC chromatograms from the purification of salvianic acid 32
A2. 1HNMR spectrum of salvianic acid 54
A3. HSQC-HMBC spectrum of salvianic acid 54
B. Stern-Volmer analysis of the fluorescence spectroscopic data 76
C. Characterization of the BSA interaction with rosmarinic, caffeic and salvianic acid through STD and NOESY NMR. 76
C1. Epitope mapping of the BSA interaction with caffeic and salvianic acid 76
C1a. Epitope mapping of the BSA-caffeic acid interaction 76
C1b. Epitope mapping of the BSA-salvianic acid interaction 76

Formatted: Font: Bold
Formatted: Hyperlink, Font: Bold, English (United States), Check spelling and grammar
Formatted: Font: Bold
Formatted: Hyperlink, Font: Bold, English (United States), Check spelling and grammar
Formatted: Font: Bold
Formatted: Hyperlink, Font: Bold, English (United States), Check spelling and grammar
Formatted: Font: Bold
Formatted: Font: Bold
Formatted: TOC 1
Formatted: Default Paragraph Font, Greek, Check spelling and grammar

C2. NOESY spectra of rosmarinic, caffeic and salvianic acid in the presence or absence of BSA 87
C2a. NOESY spectra of rosmarinic acid 98
C2b. NOESY spectra of caffeic acid 109
C2c. NOESY spectra of salvianic acid 1110
C3. STD NMR data of competitive experiments 1312
C3a. STD NMR spectra and STD amplification factors of warfarin 1312
C3b. STD NMR spectra and STD amplification factors of ibuprofen 1413
B3c. STD amplification factors of rosmarinic acid in the presence of warfarin/ibuprofen 1413
C3d. STD amplification factors of caffeic acid in the presence of warfarin/ibuprofen 1514
C3e. STD amplification factors of salvianic acid in the presence of warfarin/ibuprofen 1514
D. Isothermal titration calorimetry 1716
E. Molecular Docking to the main active sites of BSA 1817
E1. Interaction of rosmarinic acid with Sudlow site I 1918
E3. Interaction of salvianic acid with Sudlow site I 1918
E4. Interaction of warfarin with Sudlow site I 2019
E5. Interaction of ibuprofen with Sudlow site II 2120
~~A. Characterization of salvianic acid .... 2~~

Formatted: Font: (Default) +Body (Calibri)

A. Characterization of salvianic acid

A1. HPLC chromatograms from the purification of salvianic acid

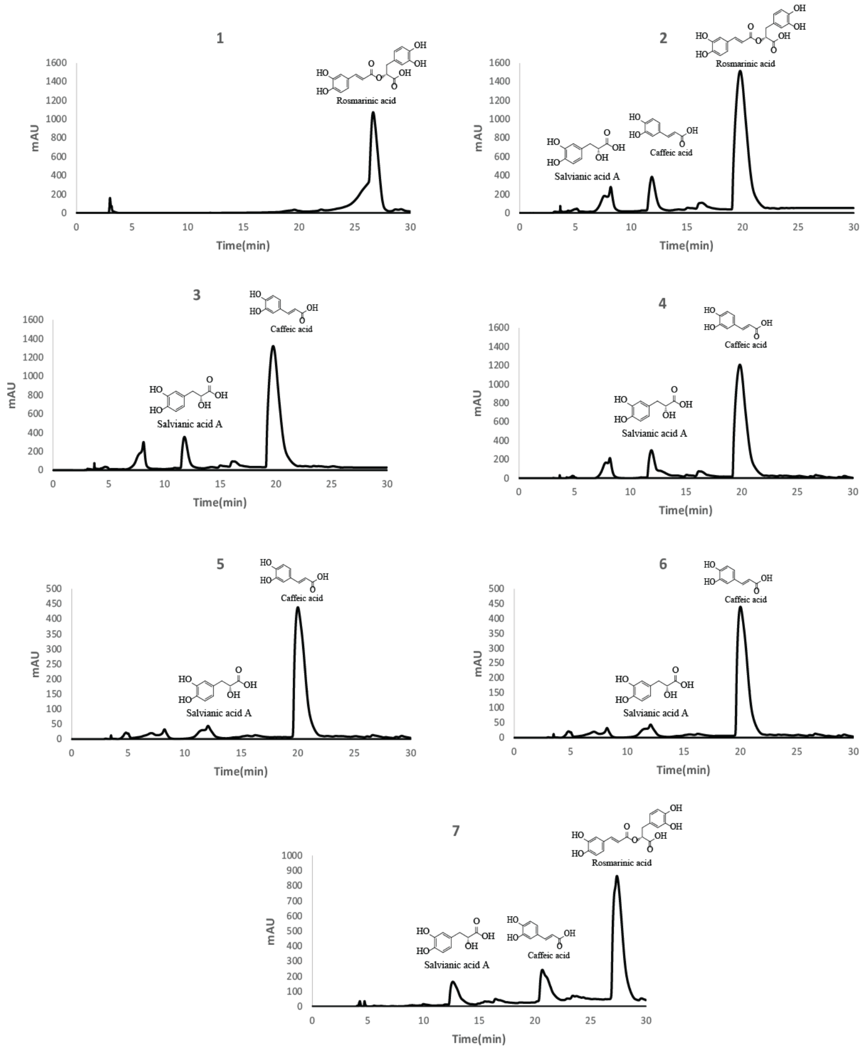

Figure S1. HPLC chromatograms from the purification of salvianic acid. (1): NaOH 0.1 M, 1 h; (2): NaOH 1 M, 4 h; (3): NaOH 1 M, 8 h; (4): NaOH 1 M, 12 h; (5): NaOH 1 M, 4 h; (6): NaOH 1 M, 8 h; (7): NaOH 0.1 M, 4h; The desired product's elution time is at 12.6 min, caffeic acid is eluted at 21 min and non-hydrolyzed rosmarinic acid is eluted at 27 min with gradient elution system MeOH–H_2O containing 0.1percent TFA (40-100% MeOH).

A2. ^{1}HNMR spectrum of salvianic acid

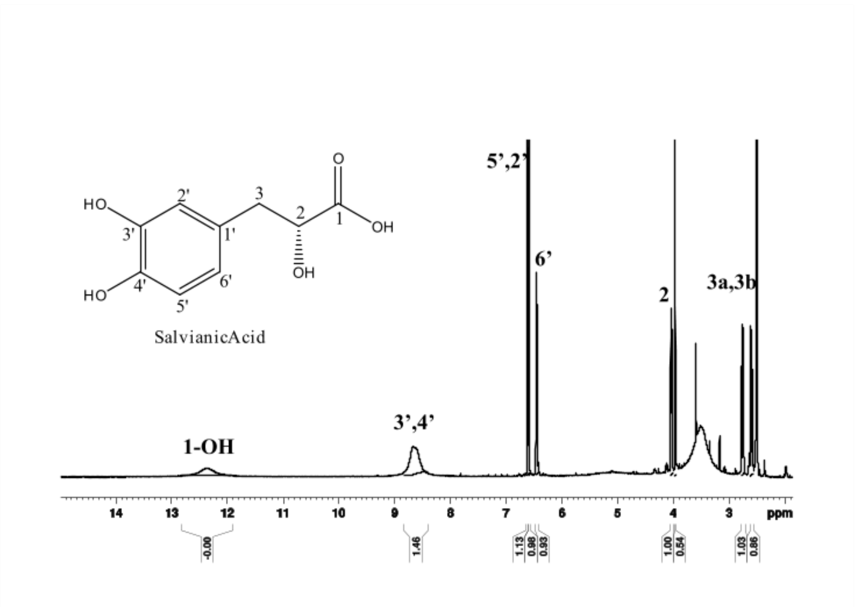

Figure S2. ^{1}HNMR spectrum of salvianic acid in DMSO-d_6, 298K, recorded at 500 MHz.

A3. HSQC-HMBC spectrum of salvianic acid

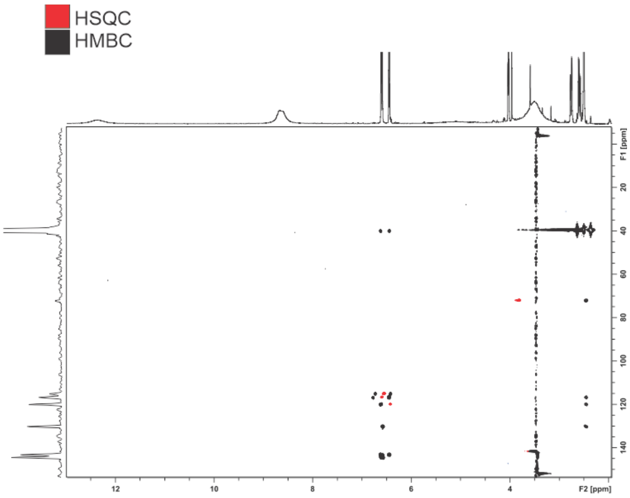

Figure S3. HSQC-HMBC overlay of salvianic acid in DMSO-d_6, 298K, recorded at 500 MHz.

B. Stern-Volmer analysis of the fluorescence spectroscopic data

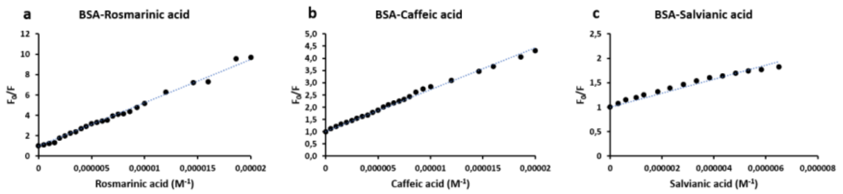

Figure S34. F_0/F as a function of A) Rosmarinic B) Caffeic and C) Salvianic acid concentration for the calculation of the Stern–Volmer quenching constant, K_{SV} at room temperature.

Table S1. The quenching constants of BSA in the presence of rosmarinic, caffeic and salvianic acid.

Compound	K_{SV} (10^4 L mol^{-1})	K_q (10^{13} L mol^{-1} s^{-1})	R^2
Rosmarinic acid	42.4	4.2	0.99
Caffeic acid	17.1	1.7	0.99
Salvianic acid	14.3	1,4	0.95

Formatted: Subscript
Formatted: Centered
Formatted: Not Superscript/ Subscript
Formatted: Superscript
Formatted Table
Formatted: Superscript
Formatted: Not Superscript/ Subscript
Formatted: Subscript
Formatted: Superscript
Formatted: Superscript
Formatted: Superscript
Formatted: Superscript
Formatted: Centered
Formatted: Centered
Formatted: Centered

~~B~~C. Characterization of the BSA interaction with rosmarinic, caffeic and salvianic acid through STD and NOESY NMR.

~~B1~~C1. Epitope mapping of the BSA interaction with caffeic and salvianic acid

~~B1a~~C1a. Epitope mapping of the BSA-caffeic acid interaction

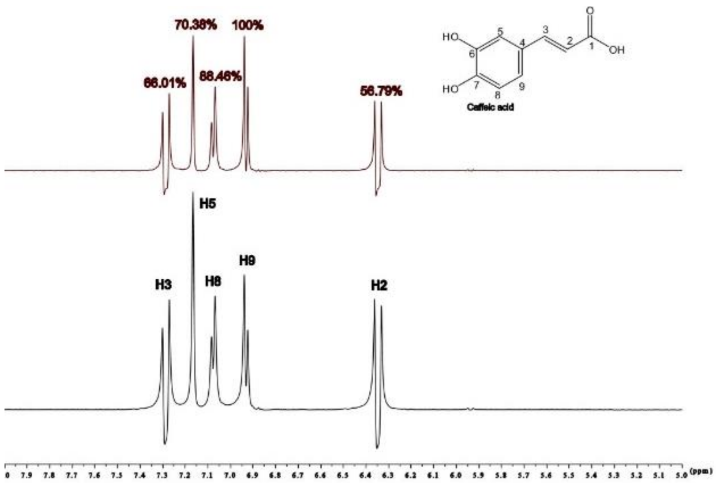

Figure ~~S4~~S5. (a) ^{1}H NMR reference spectrum of the complex caffeic acid (2 mM)-BSA (20 µM) in Tris-d_{11} buffer 10 mM, pH=7.4 with 600 µl D_2O. **(b)** STD difference NMR spectrum of the complex caffeic acid-BSA. The percentage values show the STD_{AMP} for all the protons of caffeic acid.

~~B1b~~C1b. Epitope mapping of the BSA-salvianic acid interaction

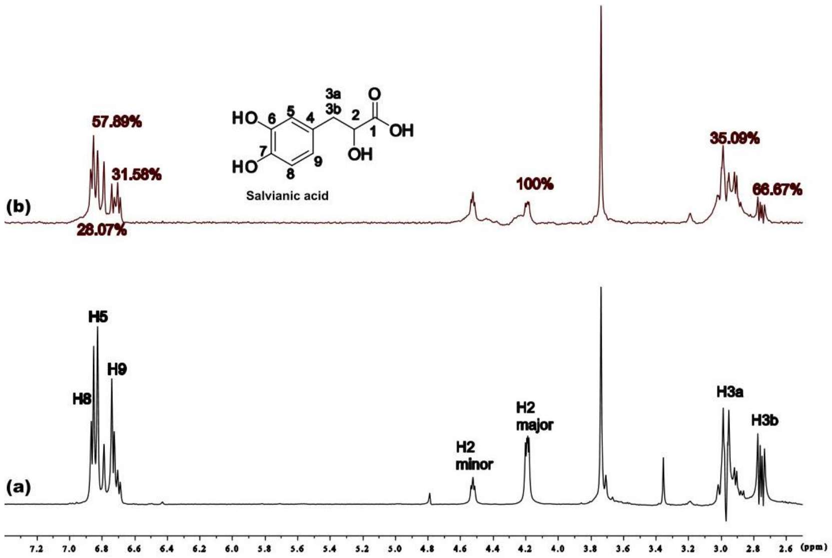

Figure ~~S5~~S6. (a) ^{1}H NMR reference spectrum of the complex salvianic acid (2 mM)-BSA (20 μM) in Tris-d_{11} buffer 10 mM, pH=7.4 with 600 μL D_2O. **(b)** STD difference NMR spectrum of the complex salvianic acid-BSA. The percentage values show the STD_{AMP} for all the protons of salvianic acid.

~~B2~~C2. NOESY spectra of rosmarinic, caffeic and salvianic acid in the presence or absence of BSA

~~B2a~~C2a. NOESY spectra of rosmarinic acid

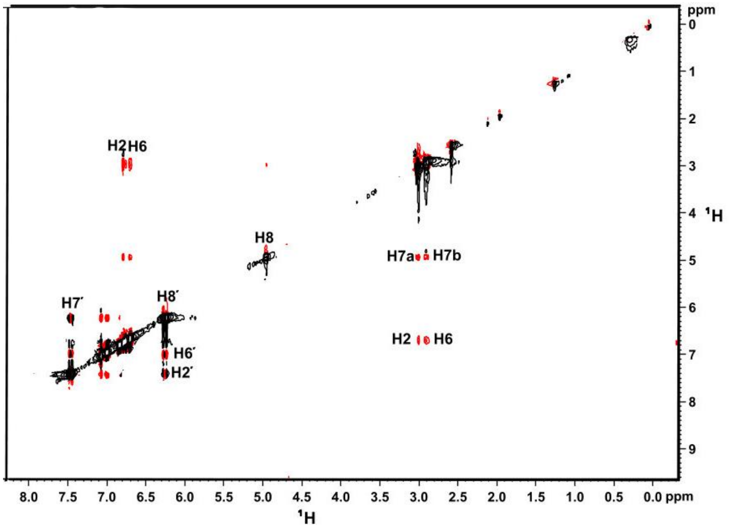

Figure ~~S6~~S7. Expanded region of NOESY spectrum of the rosmarinic acid, recorded with mixing time of 800 ms at 500 MHz, showing the presence of ~~negative~~ positive NOEs of free ligand (different sign than the diagonal).

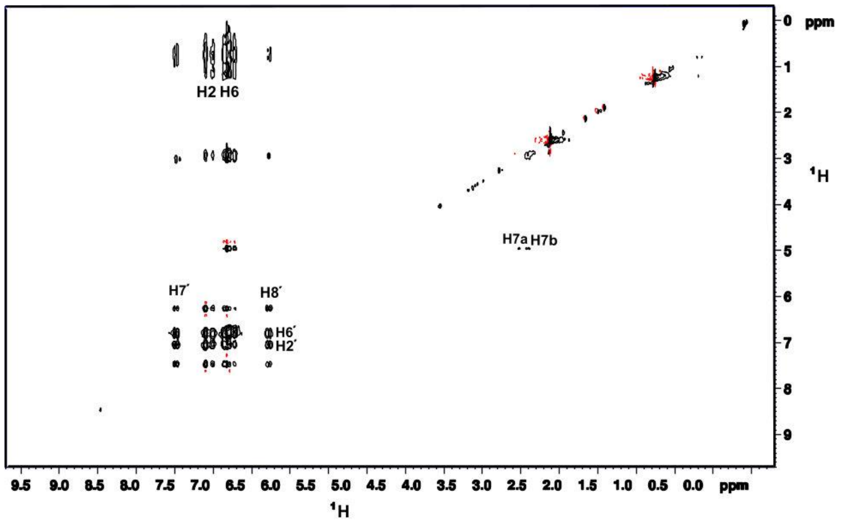

Figure ~~S7~~S8. Expanded region of tr- NOESY spectrum of the complex rosmarinic acid-BSA, recorded with mixing time of 800 ms at 500 MHz, showing the presence of ~~positive~~ negative NOEs of bound ligand (same sign as the diagonal).

~~B2b~~C2b. NOESY spectra of caffeic acid

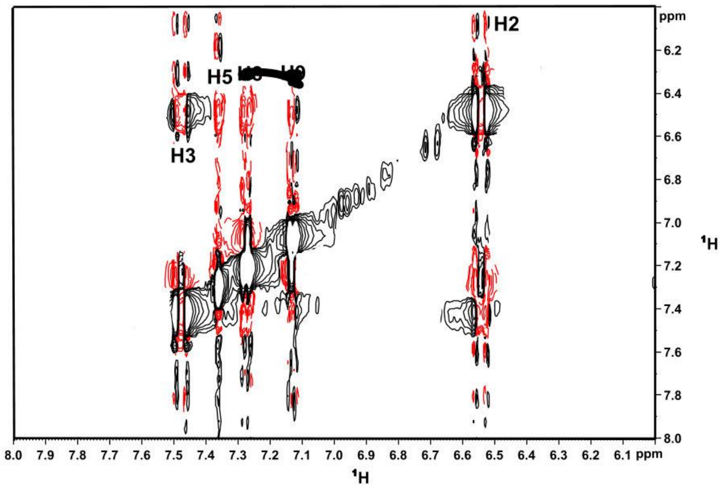

Figure ~~S8~~S9. Expanded region of NOESY spectrum of the caffeic acid, recorded with mixing time of 800 ms at 500 MHz, showing the presence of ~~negative~~ positive NOEs of free ligand (different sign than the diagonal).

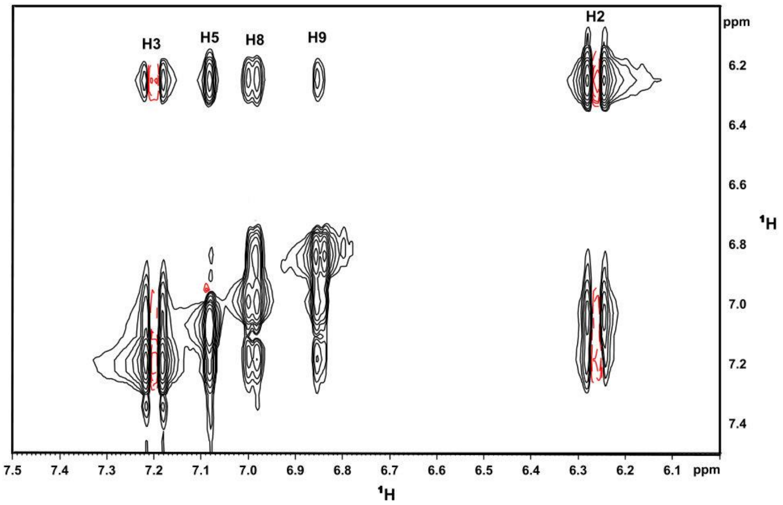

Figure ~~S9~~S10. Expanded region of tr- NOESY spectrum of the complex caffeic acid-BSA, recorded with mixing time of 800 ms at 500 MHz, showing the presence of ~~positive~~ negative NOEs of bound ligand (same sign as the diagonal).

~~B2c~~C2c. NOESY spectra of salvianic acid

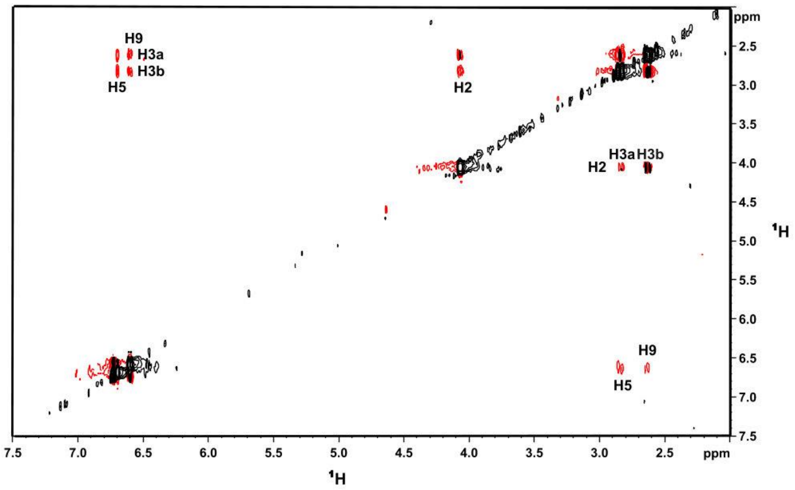

Figure ~~S10~~S11. Expanded region of NOESY spectrum of the salvianic acid, recorded with mixing time of 800 ms at 500 MHz, showing the presence of ~~negative~~ positive NOEs of the free ligand (different sign than the diagonal).

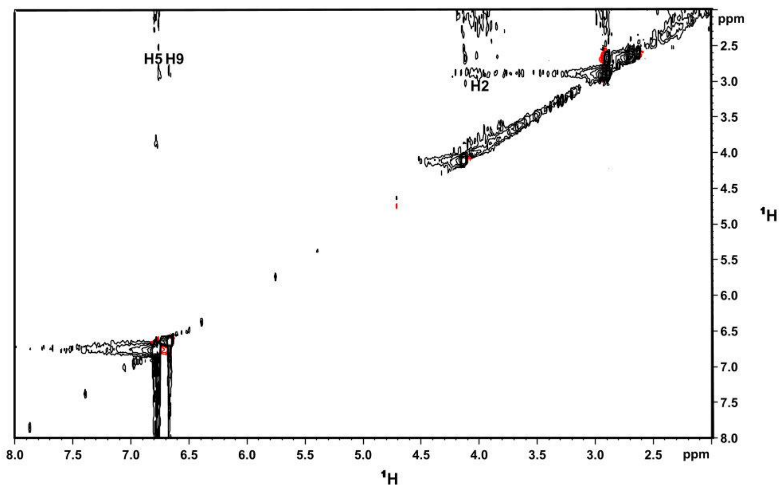

Figure ~~S11~~S12. Expanded region of tr- NOESY spectrum of the complex salvianic acid-BSA, recorded with mixing time of 800 ms at 500 MHz, showing the presence of ~~positive~~ negative NOEs of bound ligand (same sign as the diagonal).

~~B3~~C3. STD NMR data of competitive experiments

~~B3a~~C3a. STD NMR spectra and STD amplification factors of warfarin

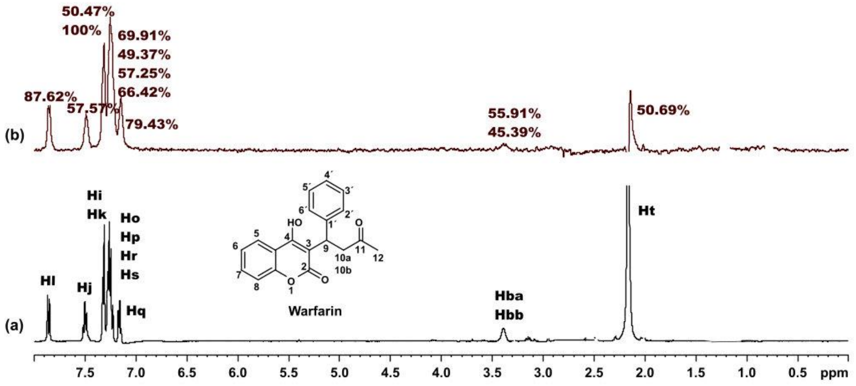

Figure ~~S12~~S13. (a) ^{1}H NMR reference spectrum of the complex warfarin (2 mM)-BSA (50 μM) in PBS buffer 10 mM, pH=7.4 with 600 μl D_2O. **(b)** STD difference NMR spectrum of the complex warfarin-BSA.

~~B3b~~C3b. STD NMR spectra and STD amplification factors of ibuprofen

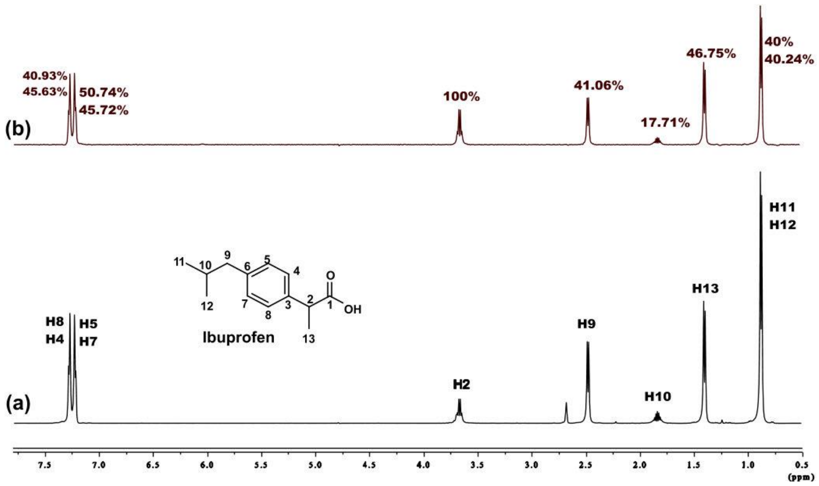

Figure ~~S13~~S14. **(a)** ^{1}H NMR reference spectrum of the complex ibuprofen (2 mM)-BSA (20 μM) in Tris-d_{11} buffer 10 mM, pH=7.4 with 600 μL D_2O. **(b)** STD difference NMR spectrum of the complex ibuprofen-BSA.

B3c. STD amplification factors of rosmarinic acid in the presence of warfarin/ibuprofen

Table ~~S1~~S2. STD_{AMP} factors occurring from the titrations of rosmarinic acid with warfarin.

	**STDAMP factors for Rosmarinic Acid**				
**Proton/Concentration**	**0 mM warfarin**	**2 mM warfarin**	**4 mM warfarin**	**6 mM warfarin**	**8 mM warfarin**
H7´	27.32%	26.54%	24.87%	22.32%	20.85%
H5´	22.25%	21.32%	19.98%	17.65%	16.39%
H2´	27.45%	26.39%	22.88%	23.33%	18.91%
H6´	22.65%	21.31%	20.83%	19.47%	18.09%
H2	34.87%	34.60%	28.91%	22.87%	22.01%

H5	19.09%	18.09%	14.51%	13.80%	13.31%
H6	24.20%	23.53%	17.66%	15.57%	7.79%
H8	4.12%	4.08%	0	0	0
H7a	12.32%	11.87%	5.11%	4.91%	3.35%
H7b	20.32%	19.85%	12.99%	11.98%	9.86%

Table ~~S2~~S3. STD_{AMP} factors occurring from the titrations of rosmarinic acid with ibuprofen.

Protons/Concentration	STD_{AMP} of Rosmarinic acid		
	**0 mM ibuprofen**	**2 mM ibuprofen**	**4 mM ibuprofen**
H7´	1.30%	2.86%	2.94%
H5´	4.20%	5.41%	6.08%
H2´	2.91%	3.55%	3.98%
H6´	3.05%	3.31%	3.40%
H8´	1.90%	2.54%	2.65%
H2	4.10%	4.51%	5.06%
H5	2.09%	2.44%	2.53%
H6	3.20%	3.46%	3.99%
H8	0.08%	0.11%	0.15%
H7a	0.21%	0.54%	0.82%
H7b	0.86%	1.04%	1.39%

~~B3d~~C3d. STD amplification factors of caffeic acid in the presence of warfarin/ibuprofen

Table ~~S3~~S4. STD_{AMP} factors occurring from the titrations of caffeic acid with warfarin.

	STDAMP factors for Rosmarinic Acid				
**Proton/Concentration**	**0 mM warfarin**	**2 mM warfarin**	**4 mM warfarin**	**6 mM warfarin**	**8 mM warfarin**
H3	26.43%	25.7%	22.68%	18.32%	4.95%
H5	26.43%	25.19%	10.68%	9.80%	3.99%
H8	12.65%	11.44%	9.41%	8.21%	8.18%
H9	14.87%	13.15%	11.19%	11.14%	9.02%
H2	15.32%	14.44%	4.95%	4.68%	4.37%

Table ~~S4~~S5. STD_{AMP} factors occurring from the titrations of caffeic acid with ibuprofen.

Protons/Concentration	STD_{AMP} factors for Caffeic acid		
	**0 mM ibuprofen**	**2 mM ibuprofen**	**4 mM ibuprofen**
H3	11.48%	12.43%	23.10%
H5	19.65%	20.17%	21.62%
H8	30.54%	31.63%	38.82%
H9	25.76%	28.83%	35.23%
H2	22.87%	23.40%	38.41%

~~B3e~~C3e. STD amplification factors of salvianic acid in the presence of warfarin/ibuprofen

Table ~~S5~~S6. STD_{AMP} factors occurring from the titrations of salvianic acid with warfarin.

	STD_{AMP} factors for Salvianic acid		
**Proton/Concentration**	**0 mM warfarin**	**2 mM warfarin**	**4 mM warfarin**
H8	25.11%	0	0
H5	27.94%	0	0
H9	30%	0	0
H2	33.78%	0	0
H3a	13.49%	0	0

Table ~~S6~~S7. STD_{AMP} factors occurring from the titrations of salvianic acid with ibuprofen.

	STD_{AMP} factors for Salvianic acid		
**Protons/Concentration**	**0 mM ibuprofen**	**2 mM ibuprofen**	**4 mM ibuprofen**
H3a	0.19%	0.26%	0.46%

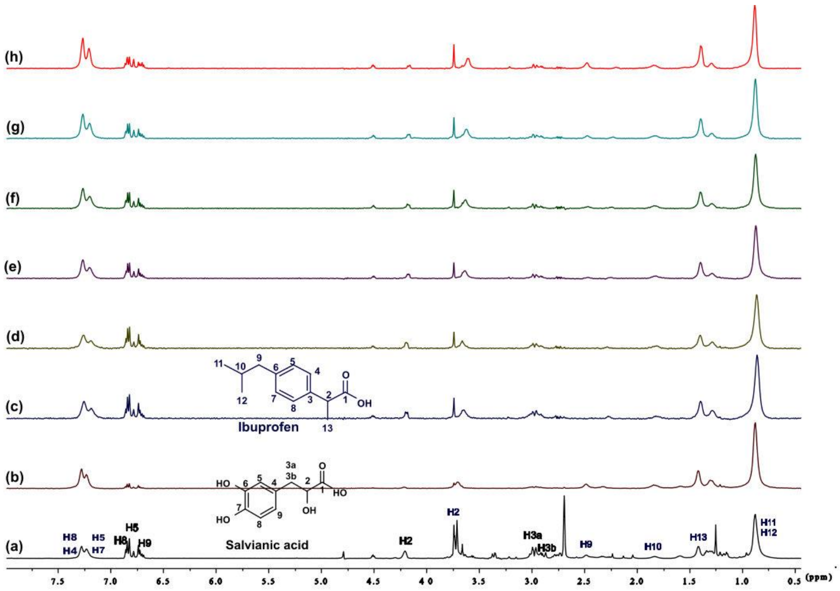

Figure ~~S14~~S15. (a) ^{1}H NMR reference spectrum of the complex ibuprofen (2 mM)-BSA (20 μM), including salvianic acid 0.2 mM, in Tris-d_{11} buffer 10 mM, pH=7.4 with 600 μl D_2O. STD difference NMR spectrum of the complex ibuprofen-BSA, including:

(b) 0.2 mM salvianic acid **(c)** 0.4 mM salvianic acid **(d)** 0.8 mM salvianic acid **(e)** 1 mM salvianic acid **(f)** 1.5 mM salvianic acid **(g)** 2 mM salvianic acid **(h)** 3 mM salvianic acid.

Table ~~S7~~S8. STD_{AMP} factors occurring from the titrations of the complex ibuprofen-BSA with salvianic acid.

	**STD_{AMP} of Salvianic acid**						
**Proton/Concentration**	**0.2 mM**	**0.4 mM**	**0.8 mM**	**1 mM**	**1.50 mM**	**2 mM**	**3 mM**
H8	0.03%	0.06%	0.10%	0.14%	0.17%	0.19%	1.31%
H5	0.05%	0.07%0.	0.10%	0.16%	0.21%	0.22%	1.63%
H9	0.05%	0.06%	0.09%	0.14%	0.19%	0.20%	1.18%
H2 minor	0%	0.08%	0.13%	0.17%	0.23%	0.23%	2.64%
H2 major	0%	0.03%	0.09%	0.10%	0.13%	0.15%	1.17%
H3a	0.01%	0.02%	0.04%	0.04%	0.10%	0.36%	0.96%
H3b	0.01%	0.02%	0.02%	0.07%	0.11%	0.15%	0.83%

Graph S1. STD_{AMP} factor values of representative protons of Ibuprofen, from the complex Ibuprofen (2 mM)- BSA (20 μM), which is titrated with salvianic acid.

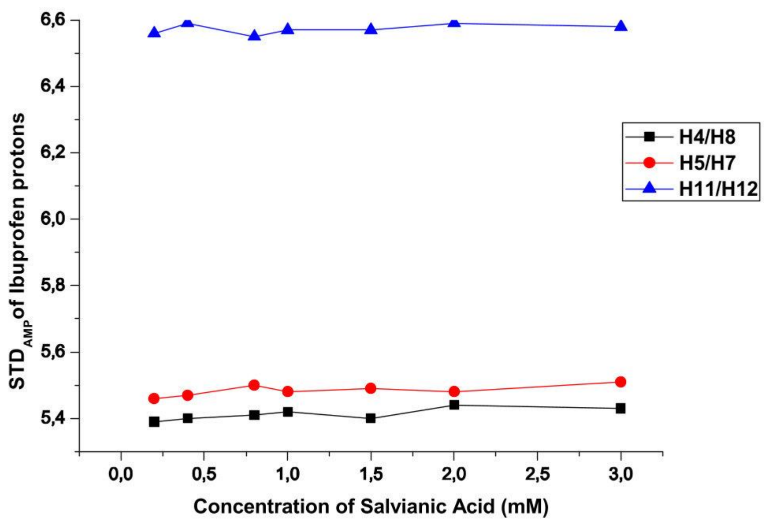

~~C~~D. Isothermal titration calorimetry

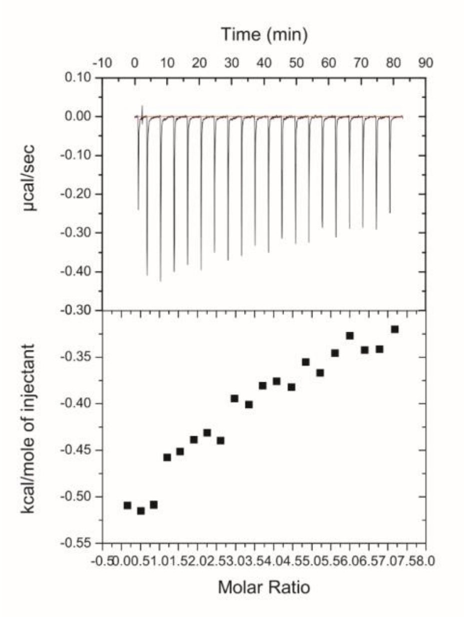

Figure S15S16. Isothermal titration calorimetry measurement for the interaction of BSA with Salvianic acid. The isotherm plot (up) and the integrated curve (down) are presented.

DE. Molecular Docking to the main active sites of BSA

Table S8S9. Binding energies of the studied molecules in Sudlow site I in BSA.

**Bovine Serum Albumin (PDB : 4F5S)**	**Sudlow Site I – Site IIA Docking Score (kcal/mol)**
Rosmarinic acid	-13.545
Caffeic acid	-11.583
Salvianic acid	-9.849
Warfarin	-9.922
Ibuprofen	-6.048

~~D1~~E1. Interaction of rosmarinic acid with Sudlow site I

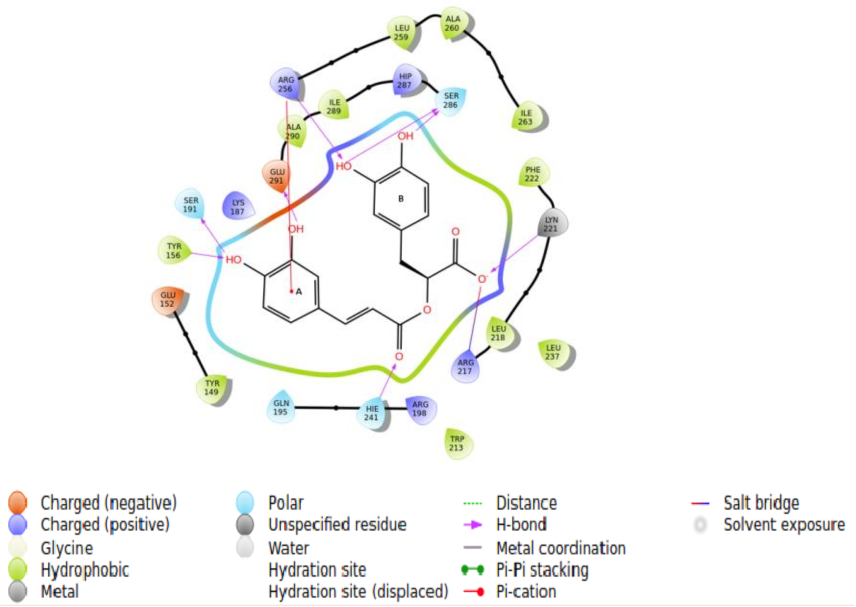

Figure ~~S16~~S17. Binding interactions of rosmarinic acid in Sudlow site I of BSA.

~~D3~~E3. Interaction of salvianic acid with Sudlow site I

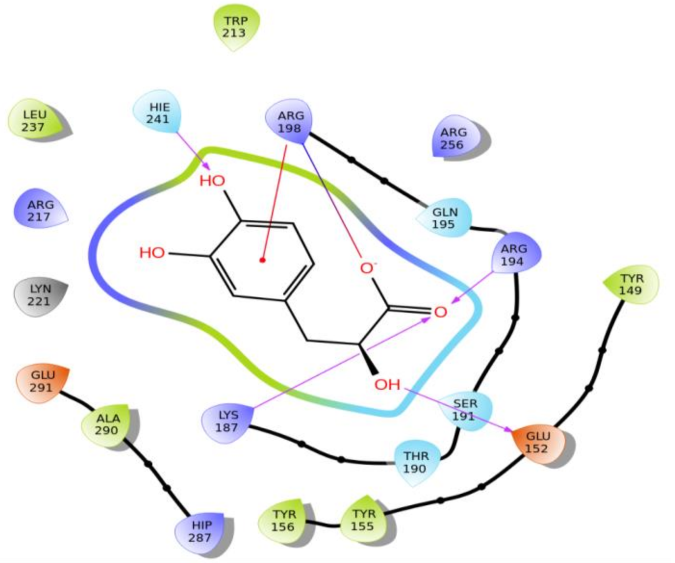

Figure ~~S17~~S18. Binding interactions of salvianic acid in Sudlow site I of BSA.

~~D4~~E4. Interaction of warfarin with Sudlow site I

Warfarin shows a very favored binding value (Docking Score = -9.922 kcal / mol) in Sudlow site I (**Table ~~S8~~S9**). Warfarin in its best pose conformation (**Figure ~~S18~~S19, ~~S19~~S20**) forms H – bonds with four residues of the protein (Arg194, Arg198, His241, Arg256). In addition, the formation of a salt bridge between Arg217 and the enol group of the ligand contributes to the stabilization of the molecule in the binding site. Importantly, there are strong coulombic interactions between Sudlow site I and warfarin and this makes the molecule to adopt preferable binding to this site.

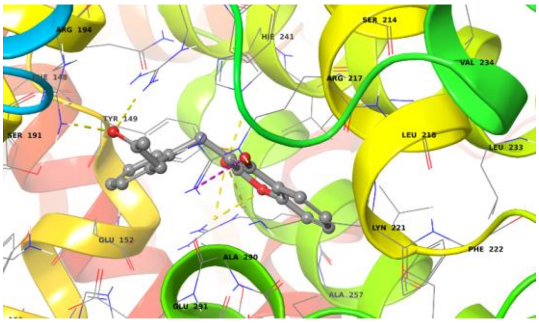

Figure ~~S18~~S19. Best pose of warfarin in Sudlow site I. The four favorable hydrogen bonds, the salt bridge between Arg217 and the enol group of the ligand can explain its highly favorable binding.

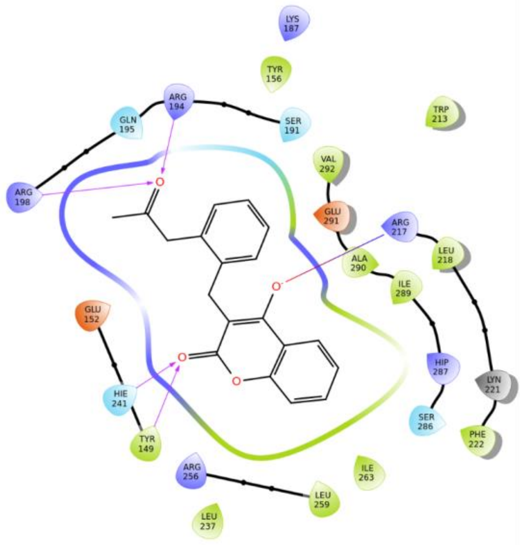

Figure ~~S19~~S20. Binding interactions of warfarin in Sudlow site I of BSA.

~~D5~~E5. Interaction of ibuprofen with Sudlow site II

Docking calculations, using empirical scoring functions, expressed a value of free energy binding in Sudlow site II equal to -9.769 kcal / mol (**Table ~~S8~~S9**). Ibuprofen

in its best pose conformation (**Figure** ~~S20~~S21, ~~S21~~S22) forms H – bonds with three residues of the protein (Ser488, Asn390, Arg409). In addition, the formation of the salt bridge between the carboxylic group of the ligand and Lys413 plays a key – role in the stabilization of the complex. In the low value of free energy, the orientation of the aromatic ring towards the hydrophobic residues (i.e. Leu452, Leu456, Leu406, Leu429, Val432, Cys391, Cys436, Cys437, Phe394, Phe402) plays an important role too.

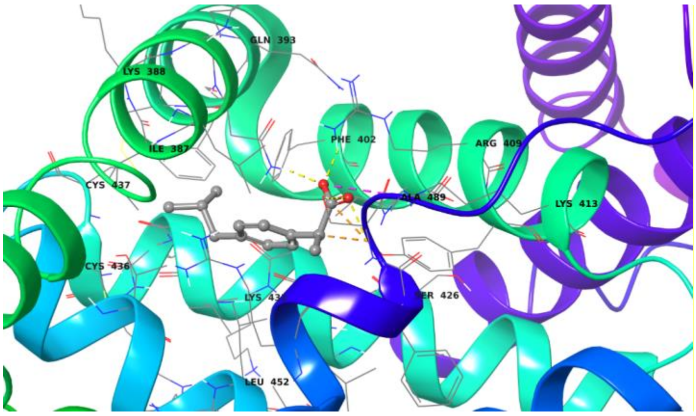

Figure ~~S20~~S21**.** Best pose of ibuprofen in Sudlow site II. The three favorable hydrogen bonds and the salt bridge formed between Lys413 and the carboxylic group of the ligand can explain its highly favorable binding.

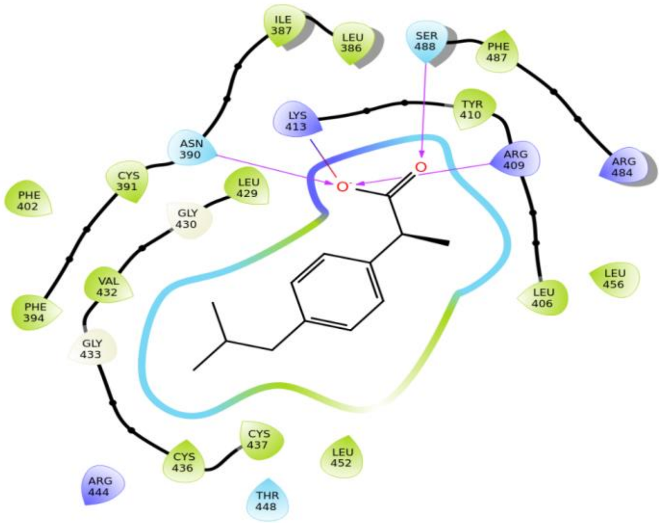

Figure ~~S21~~S22. Binding interactions of ibuprofen in Sudlow site II of BSA.
